# Patterns of genetic diversity in North Africa: Moroccan-Algerian genetic split in *Juniperus thurifera* subsp. *africana*

**DOI:** 10.1038/s41598-020-61525-x

**Published:** 2020-03-16

**Authors:** Asma Taib, Abdelkader Morsli, Aleksandra Chojnacka, Łukasz Walas, Katarzyna Sękiewicz, Adam Boratyński, Àngel Romo, Monika Dering

**Affiliations:** 1 Laboratoire de Ressources Génétiques et Biotechnologies, Ecole Nationale Supérieure Agronomique, Avenue Pasteur, Hassan Badi, 16200 Algiers, El Harrach Algeria; 2 Institute of Dendrology Polish Academy of Sciences, Parkowa 5, 62-035 Kórnik, Poland; 3 Botanical Institute of Spanish Research Council, Passeig del Migdia, s/n, 08038 Barcelona, Spain; 4 Department of Forest Silviculture, Poznań University of Life Sciences, Wojska Polskiego 71A, 60-637 Poznań, Poland

**Keywords:** Ecology, Biogeography, Evolution, Population genetics

## Abstract

*Juniperus thurifera* is a key element of the forest communities in arid and semi-arid areas of the western Mediterranean. Previous genetic and morphological investigations suggested that Algerian populations are genetically more similar to European than to Moroccan populations and advocated their recognition at the variety rank. We aimed to investigate the spatial genetic structure in *J. thurifera* to verify the distinct character of the Algerian population in terms of the genetic breaks reported among several North African taxa. We also modelled species distributions since the Eemian to recognise the impact of past climatic changes on the current pattern of diversity and predict possible changes in species distribution in the future. Species-specific microsatellites were used in the analysis of 11 populations from Algeria, Morocco and Europe. We revealed the significant genetic distinctiveness of the Algerian populations from the Moroccan and European stands that may have important taxonomic and conservation implications. The diversity pattern revealed for *J. thurifera* reflects the east-west genetic splits reported among some North African plant and animal taxa and suggests an impact of shared historical processes. Additionally, modelling of the distribution allowed us to identify possible glacial refugia and their impact on the modern pattern of differentiation in *J. thurifera*. Reduction of species occurrence, especially in the European domain, is likely according to the future projections of the species distribution.

## Introduction

Compared to the northern Mediterranean, the genetic diversity and differentiation patterns of woody species in the southern part of the region that comprises North Africa is rather poorly explored^1,2^. However, in some studies that were focused on the evolutionary history of the North African tree species, the distinctiveness between Moroccan and Algerian/Tunisian populations emerges recurrently. In investigations on the cpDNA variability of *Quercus canariensis*^3^ and *Q. ilex*^4^ and mtDNA diversity of *Pinus pinaster*^5^, the Algerian populations were shown to have different haplotypes from those occurring in Morocco; a similar conclusion was drawn for *Olea europea*^6^. Furthermore, Moroccan stands of *Cedrus atlantica* and *Alnus glutinosa* are also genetically distinct from the Algerian populations^7,8^. The divergence revealed in *A. glutinosa* also included differences in ploidy level, such as Moroccan species displaying tetraploidy in comparison to the diploidy displayed by Algerian and European populations. Phylogeographic parallels among plants are accompanied by numerous examples among animal species^9–12^, and both advocate for a more general hypothesis of the phylogeographic convergence among North African biota. Such a pattern would assume an east-west split among North African biota, implying a common historical process in action.

*Juniperus thurifera* L. (thuriferous juniper) occurs in Europe and North Africa but the species range is greatly fragmented^13–17^. The major distribution area covers the mountains of the Iberian Peninsula, followed by the areas of the High and Middle Atlas Mountains, with a separate subpopulation in the Anti-Atlas in Morocco^14,18–20^. The species distribution has even been observed to extend into Spain through colonization of abandoned fields and abandonment of livestock operations^21^. Beyond the Iberian Peninsula, the species is considered rare in Europe, and it occurs in the Southern Alps, the Pyrenees and Corsica. In reference to the African range, the Moroccan populations are extremely degraded^18,22^, but the most isolated and confined populations of this species are in Algeria^22^.

In Algeria, *J. thurifera* is exclusively found in six locations in the Aurès Mountains, which are the eastern continuation of the Saharan Atlas Mountain range and are considered to be one of the 39 key biodiversity areas in northern Algeria^23^. The areas with the most inventories indicated the existence of relatively dense stands consisting of 3,500 to 8,000 mature individuals^24^. In addition to the climate-driven contraction of juniper’s range in Africa, its decline is strictly related to human pressure. First, it has declined as a result of overexploitation due to logging and wood harvesting, because juniper wood has been used in North Africa as a fuel for the last several millennia^16^. Second, maintaining a high level of livestock and traditional pasture culture in Mediterranean countries, which results in overgrazing, is also an important factor affecting the species decline in Algeria and Morocco^25^. Overgrazing and branch cutting negatively affect the regeneration ability of this species, since the organic matter that accumulates beneath the trees’ crowns is the most suitable site for recruitment^16^. A lack of such conditions hampers regeneration and leads to demographic collapse. Currently, all Algerian stands suffer from a lack of natural regeneration, and old individuals prevail in most of the populations, which probably also decreases their reproductive abilities. Generally, thuriferous juniper in Algeria produces seeds sporadically, and most of them are empty (Taib, personal observation), which suggests the occurrence of some serious environmental or genetic factors (or likely both) that interfere with the reproduction process, causing its failure. Additionally, in all stands, an imbalanced sex ratio is noted, and female trees represent a maximum of 20% of the population size. Since recruitment success is highest under female trees, an insufficient number of such trees in a population is another factor that negatively affects the regeneration process^26^.

The highly fragmented distribution of *J. thurifera* corresponds to its morphological and genetic differentiation, which led to subdivision into intra-specific taxa. The major taxonomic split follows the Ibero-African species distribution and resembles the isolating effect of the Strait of Gibraltar^27–29^. The morphological and genetic distinctiveness of populations from Europe and North Africa led to the recognition of the two subspecies: *J. thurifera* subsp. *thurifera* and *J. thurifera* subsp. *africana* (Maire) Romo et Boratyński^20,30–34^. Within the European species range, the geographic varieties are described, namely, *J. thurifera* var. *gallica* in the Alps and the Pyrenees, *J. thurifera* var. *hispanica*^18^ in Spain and *J. thurifera* var (chimovar.) *corsicana* Gauquelin, Idr. Hass. & P. Lebreton in Corse^35^. A suggestion to separate the Algerian relict populations under the name *J. thurifera* var. *aurasiaca* Véla & P. Schäf., *var. nov*. was made by Vela and Schäfer^35^ based on the geographic and morphological criteria. The distinctiveness of the Algerian stands was confirmed by Zeraib *et al*.^36^ in chemotaxonomic studies. However, the intermediate genetic character of a single Algerian population analysed by Terrab *et al*.^37^ suggested that it was more closely genetically related to the European stands.

According to the International Union for Conservation of Nature (IUCN), *J. thurifera* has been classified as being of *least concern*, which may be reasonable in the case of Spanish populations that occupy large areas^38^. However, in North Africa, species exhibits population fragmentation, reduced regeneration and strong decline in Algeria^39^. Particularly, investigations of Algerian populations are important because they may deliver information about the species’ genetic resources and reveal whether those populations form a distinct genetic lineage that should be recognized as a separate conservation unit. The very marginal location of the Algerian populations in the Aurès Mountains that practically precludes gene flow from other parts of the species’ geographic range may pose a threat to its long-term survival, especially in terms of ongoing climate change^40^. Moreover, genetic responses to climate-driven environmental changes will likely be based on standing genetic diversity, which requires the maintenance of high within-population variability^41^.

In this paper, genetic analysis was undertaken to examine the differences between the Algerian populations of *J. thurifera* in reference to the Moroccan and European stands, as previous studies were unconvincing^37^. We then asked two questions: (i) Does the spatial pattern of the genetic diversity of *J. thurifera* subsp. *africana* match the east-west genetic breaks reported for some North African organisms? and (ii) Does the intraspecific genetic differentiation have taxonomic and hence conservation implications for the Algerian populations? To predict the impact of climate change and evaluate the potential risks involved, species distribution modelling (SDM) was performed. Recently, this method has been widely used in simulations of future changes in species distribution patterns induced by climate change and has been recognized as a useful tool that may efficiently support conservation genetic studies and further planning of conservation activities^42–44^.

## Materials and methods

### Plant material

**Figure 1:**
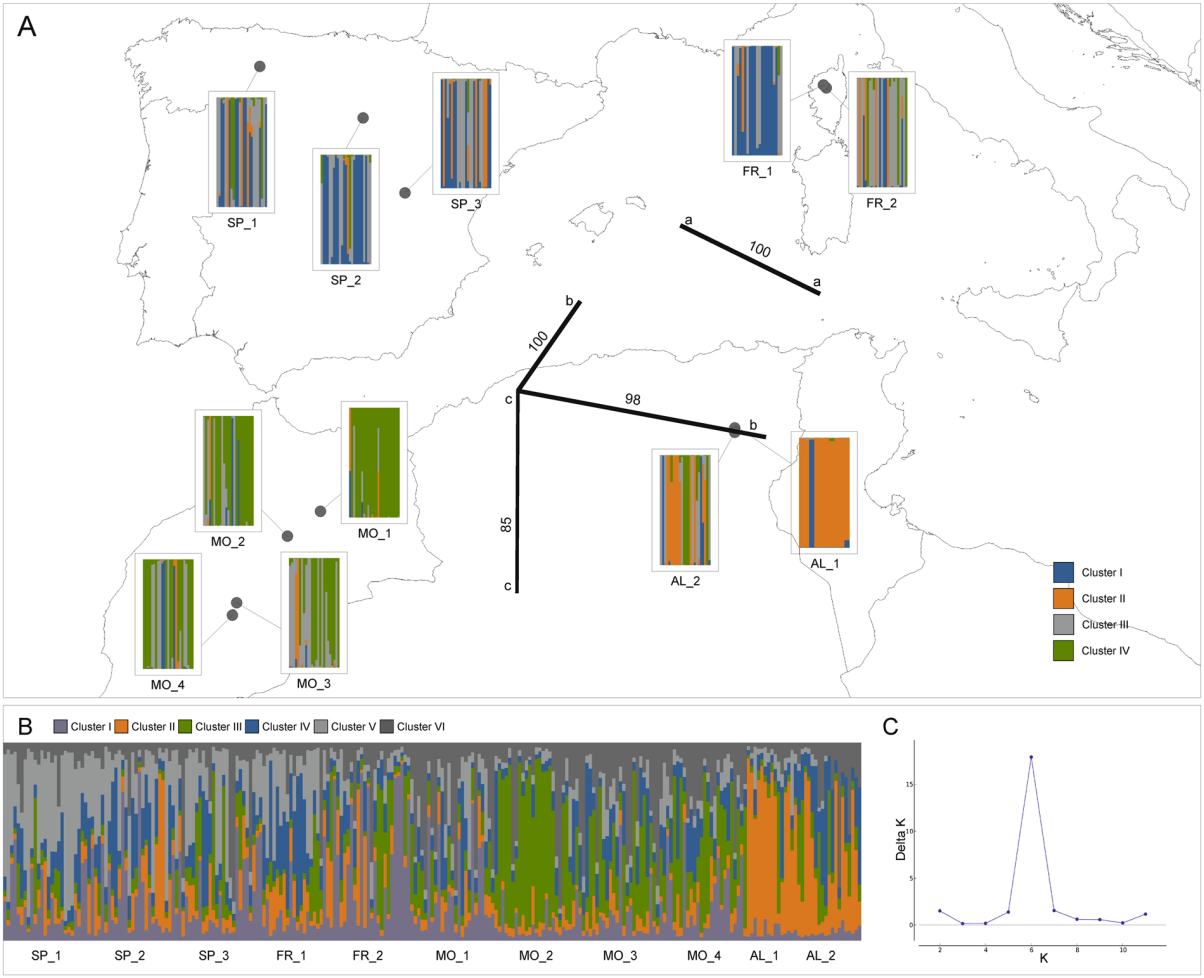
Location of the *J. thurifera* populations subjected to genetic analysis and populations genetic structure based on six nSSR loci: (**A**) – bar plots obtained from a discriminant analysis of principal components (DAPC) with genetic barriers obtained with Monmonier’s algorithm (bold lines); (**B**) – proportion of membership of each individual in the six assumed clusters (K = 6) according to a Bayesian approach estimated by STRUCTURE; (**C**) – estimation of the optimal number of genetic clusters following Evanno’s ΔK method (Evanno et al. 2005). Map prepared with QGIS.

Nine natural populations of *J. thurifera* from Spain, France (Corsica) and Morocco (Middle and High Atlas Mountains) that were previously biometrically analysed by Boratyński *et al*.^33^ and two populations from Algeria (Aurès Mountains) were used in this study for the screening of polymorphisms at nuclear microsatellite loci (nSSR) (Table 1, Fig. 1A). However, permission for material collection in Chelia and Tafrent in Algeria was very restricted, and we were able to access only a very limited number of individuals (Table 1). Leaves were collected from 10–25 randomly distributed individuals from each population; 255 individuals were sampled in total.

**Table 1:** Location of the studied populations of *J. thurifera*, summary statistic of genetic variability and AMOVA-based *k*-means clustering for the optimal number of clusters (*k* = 4 and *k* = 9).

Population ID	Population names	Location	N	N_a_	N_e_	P_a_	H_s_	AMOVA-based *k*-means	
								*k* = 4	*k* = 9
SP_1	Spain, Leon, Montes de la Luna	42.783 N 05.75 5W	25	12.33	5.48	2	0.79	1	1
SP_2	Spain, Soria, Sierra de Cabreja, slopes above Ucero	41.716 N 03.050 W	25	14.67	6.06	2	0.75	1	2
SP_3	Spain, Cuenca, Serranía de Cuenca, between La Toba and Buenache de la Sierra	40.166 N 01.699 W	25	12.83	5.54	2	0.76	1	6
FR_1	France, Corse, Niolo, Monte Cinto	42.368 N 08.963 E	21	14.17	5.91	2	0.78	1	6
FR_2	France, Corse, Calacuccia, Golo Valley	42.341N 09.003 E	25	11.83	4.89	2	0.69	2	5
		***Average***		***13.166***	***5.576***		***0.754***		
MO_1	Morocco, Middle Atlas, Jbel Bou Iblane, E of Talzemt	33.600N 04.166 W	25	13.67	5.49	0	0.76	4	8
MO_2	Morocco, Middle Atlas, Aguelmame Sidi-Ali	33.078 N 05.0250 W	25	12.50	4.90	1	0.76	4	9
MO_3	Morocco, High Atlas, Jbel Azourki, below Tizi-n-Ilissi, SE slopes above Iglaouane	31.700 N 06.349 W	25	14.83	5.64	1	0.79	4	8
MO_4	Morocco, High Atlas, slopes above Tessaout (Toufrine)	31.450 N 06.466 W	25	13.00	5.81	0	0.78	4	3
		***Average***		***13.500***	***5.460***		***0.773***		
AL_1	Algeria, Aurès Mts., Chelia	35.310 N 06.626 E	10	10.17	5.79	1	0.81	3	4
AL_2	Algeria, Aurès Mts., Tafrent	35.216N 06.622 E	24	14.83	6.18	1	0.81	3	7
		***Average***		***12.500***	***5.985***		***0.810***		

N - number of individuals; N_a_ - average number of alleles; N_e_ - effective number of alleles; P_a_ - number of private alleles; H_s_ - heterozygosity within populations.

### DNA extraction, amplification and sequencing

Genomic DNA was extracted from dried leaves using the CTAB method^45^. A set of six nSSR markers originally developed for *J. thurifera* that were highly polymorphic according to Teixeira *et al*.^34^ were used: JT_01, JT_04, JT_30, JT_33, JT_40 and JT_46 (Supplementary Table S1). Two multiplex PCRs were performed in a final volume of 10 µL containing approximately 60 ng of template DNA, 1 U of Silver Hot Start DNA Polymerase (Syngen Biotech, Poland), 1 µM of each primer pair, 1x reaction buffer, 2.5 mM MgCl_2_, and water. The first multiplex reaction involving loci JT_01, JT_30 and JT_33 and the second one with loci JT_04, JT_40 and JT_46 were amplified in a Labcycler Basic thermocycler (SensoQuest, GmbH) with the following conditions: an initial denaturation step of 15 min at 95 °C, followed by 40 cycles of denaturation at 94 °C for 30 sec, annealing at a temperature specific to each multiplex for 90 sec (57 °C for multiplex I and 55 °C for multiplex II), and extension at 72 °C for 60 sec, with a final extension step at 72 °C for 10 min. PCR products were analysed using an AB 3130 Genetic Analyzer (Thermo Fisher Scientific, USA) capillary electrophoresis system with an internal size standard, GeneScan™ 500 LIZ^®^. Genotypes were scored using GeneMapper 4.0 software (Thermo Fisher Scientific, USA).

### Data analysis

#### Genetic diversity and differentiation

*J. thurifera* is a tetraploid species^34,46^, which has important practical consequences for the scoring of genotypes and subsequent statistical analysis of nSSR data^47,48^. There is substantial ambiguity in genotype assignment due to the difficulty of resolving the number of allele copies in polyploids. This methodological issue leads to situations in which genotypes cannot be truly inferred based on phenotypes; therefore, neither allele frequencies nor genotype frequencies can be estimated, which are necessary to implement population genetic-based inferences^47,49^. Additionally, fixation of heterozygosity is a common feature, because genetically different isoloci may share the same alleles, which precludes statistical evaluation of deviation from the Hardy-Weinberg equilibrium or gene diversity level^50^.

GenoDive 2.0b23 software^51^, which enables population genetic analysis of polyploids by correcting for allele copy number ambiguity, was used for computations of basic diversity parameters, such as the number of alleles (N), average number of alleles (N_a_), effective number of alleles (N_e_), number of private alleles (P_a_) and heterozygosity within populations (H_s_). The missing alleles in genotypes were filled with the option *overall allele frequencies*. The overall genetic differentiation among populations was assessed using the fixation index, G_st_^52^, and tested with 9,999 permutations. Finally, pairwise population differentiation (F_st_) was calculated from an analysis of molecular variance (AMOVA), which is exactly equivalent to Weir and Cockerham’s^53^ statistic, and the significance of F_st_ was tested with 9,999 permutations.

#### Geographic patterns of differentiation

We applied different multivariate analysis methods for detecting population structure that can be applied to polyploids^48^. First, AMOVA-based *k*-means clustering analysis^54^ implemented in GenoDive was applied. This method uses the AMOVA framework to calculate the among-cluster sum of squares and divides individuals into an a priori assigned number (*k*) of groups in such a way that the among-cluster sum of squares is maximized based on a simulated annealing algorithm. Analysis was performed on all generated datasets for *k* values from 2 to 9, using 20 repeats of the simulated annealing algorithm, with 50,000 MCMC steps. To estimate the most likely number of clusters (optimal *k*), the Bayesian information criterion (BIC) and Caliński and Harabasz pseudo-*F* statistic^55^ methods were used. The clustering results that were biologically justified were discussed, according to the recommendation of Meirmans^54^.

Second, to infer the geographical pattern of genetic differentiation and detect potential genetic admixture, a discriminant analysis of principal components (DAPC)^56^ implemented in package ‘*adegenet*’ in R 3.4.3^57,58^ was used. This multivariate analysis provides an alternative to model-based analyses, because it is not limited by assumptions about the population genetic models; thus, it may serve as a more efficient tool for unravelling complex population structure^56^. Analysis was performed on a genetic dataset that was transformed to a binary matrix of the presence and absence of each allele without defining groups a priori. DAPC is a multivariate two-step procedure in which data are first transformed by principal component analysis (PCA) as a prior step to a discriminant analysis (DA). The function ‘*find cluster’* was used to identify the optimal number of clusters based on the BIC. The number of principal components (PCs) to be retained for the DAPC was chosen according to the α score using the ‘*optim.a.score*’ function; the DAPC was conducted based on 12 PCs.

Monmonier’s maximum-difference algorithm implemented in BARRIER 2.2^59^ was used to identify genetic discontinuities among populations. This approach was confirmed to be efficient in identification of intraspecific lineages^60^. The analysis was run using Nei’s genetic distance matrix and the geographical coordinates of each population. The significance of each inferred barrier was assessed by bootstrapping with 100 replications of the genetic distance matrix. SEQBOOT and GENDIST programmes implemented in the PHYLIP package 3.696 were used to obtain multiple matrices data for bootstrapping.

Finally, a model-based Bayesian clustering approach implemented in STRUCTURE 2.3.3^61^ was used to determine the spatial genetic structure. A non-spatial admixture model with correlated allele frequencies was used. To deal with the *4n* ploidy level that generates genotype ambiguity, we used a recessive alleles option as recommended by the software’s manual and recent paper of Meirmans^48^. Ten independent repetitions for each number of groups (K) ranging from 1 to 12 were performed with a burn-in set of 100,000 steps, followed by 200,000 MCMC iterations. The CLUMPAK platform^62^ was used to align replicated runs and average individual assignment probabilities for the most likely K-value and to estimate the optimal number of clusters according to Evanno’s ΔK method^63^.

To determine the hierarchical distribution of genetic variation, AMOVA implemented in GenoDive was performed for two defined configurations: among geographic regions (Europe, Morocco and Algeria) and groups revealed by clustering analysis conducted by DAPC *(k* = 4). The significance was tested with 9,999 permutations, and distances were calculated using the ploidy independent infinite alleles model (*Rho*). To test for isolation by distance (IBD), the geographic distance matrix was correlated with the genetic distances (F_st_) using the standard Mantel test implemented in GenoDive and significance was assessed with 9,999 permutations.

Graphical presentation of genetic data was made with using the QGIS Geographic Information System^64^.

#### Modelling of species distributions

The species distribution model for *J. thurifera* was built using the maximum entropy approach implemented in MaxEnt 3.4.1^65,66^. The data on occurrences of *J. thurifera* were obtained from the literature and personal observations (Supplementary Table S2). All data were carefully verified, and a total of 280 points were accepted for analysis^19,37,46,67^. Bioclimatic variables were retrieved from the WorldClim database^68^ and CHELSA 1.2^69^ to construct models presenting the species’ current potential distribution based on climatic suitability (*c*.1960–1990; PRE) and that at the last glacial maximum (LGM; 22 ka BP). The CHELSE database has been shown to deliver more precise estimations in terms of precipitation than WorldClim, because the precipitation estimation algorithm is based on statistical downscaling of atmospheric temperatures, which delivers improved climatologies, especially in mountainous landscapes^69^. The spatial resolution of 19 bioclimatic variables^68^ (Supplementary Table S3 and S4) used to model the current species range was 30 arc-sec, and for the LGM scenario, the Community Climate System Model (CCSM4) with a 2.5 arc-min resolution was used^70^. Additionally, to predict future distributional changes in the species’ range, the RCP 2.6 scenario of climate change was tested for the CCSM4 model at a 2.5 arc-min resolution. This scenario assumes that before the year 2100, the atmospheric CO_2_ concentration will reach 440 ppm and increase the radiative forcing by 2.6 W/m^2^ as well as that the increase in global mean surface temperature by the end of the 21st century (2081–2100) will be 0.3 °C to 1.7 °C.

The bootstrapping analysis was conducted with 100 replicates, the convergence threshold was set to 10^–5^, and the maximum number of iterations was 10,000. The ‘random seed’ option was applied, 20% of the data were set aside as test points, and the output was set to logistic. To reveal areas of long-term species persistence, such as Pleistocene refugia, we also modelled the Eemian (*c*. 120–140 ka BP) theoretical range of *J. thurifera* in Europe and Africa. However, the Eemian projection was available only with WorldClim. We performed modelling of the present and LGM distributions using occurrence data (1) jointly for African and European populations, (2) for African populations only and (3) for European populations only using both climatic datasets. As a criterion of model accuracy, the receiver operating characteristic (ROC) curve and value of the area under the curve (AUC) were used^71,72^. The results of the analyses were visualized in QGIS 2.18.20^64^.

In addition, the relative influence of each bioclimatic variable on the current distribution of *J. thurifera* was verified by PCA analysis in order to characterize ecological differences between the African and European populations. Analysis was performed on dataset generated for 583 occurrence records of species (Supplementary Table S5) based on bioclimatic raster retrieved from CHELSA using SAGA GIS software^73^. Function ‘*vif*” from package *usdm* in R was used to calculate the variance inflation factor (VIF). Ten climatic variables, which had the highest VIF values (> 300), were removed from the analysis to avoid autocorrelations. PCA analysis was run using the ‘*prcomp*’ function implemented in R.

Graphical presentation of modelling results was made using the QGIS Geographic Information System.

## Results

### Genetic diversity and differentiation

All loci used were polymorphic, and the number of alleles ranged from 12 (JT_04) to 44 (JT_33), for a total of 144 alleles detected across the 255 individuals analysed (Supplementary Table S1). In terms of allelic diversity, the Algerian populations were less variable (average N_a_ = 12.500; N_e_ = 5.985) than the European (average N_a_ = 13.166; N_e_ = 5.576) and Moroccan populations (average N_a_ = 13.500; N_e_ = 5.460) (Table 1). In all populations (except for MO_1 and MO_4), private alleles were detected, but the largest number (10) was noted in the European populations. The heterozygosity within populations (H_s_) ranged from 0.687 (FR_2) to 0.809 (AL_2), with an average value of 0.772. A comparison between the European part of the *J. thurifera* range and the African populations indicated that the population from Algeria had the highest significant average value of within-population diversity (H_s_ = 0.808; P = 0.133), but this value was not significantly higher than the other values (Table 1).

The overall genetic differentiation among populations was low but highly significant (G_st_ = 0.026; P < 0.001). Accordingly, the populations’ pairwise F_st_ values ranged from 0.002 (between populations MO_1 and MO_3) to 0.059 (between populations FR_2 and AL_1) and were significant (P ≤ 0.001) in most populations (Table 2).

**Table 2:** Pairwise estimates of F_st_ among studied populations of *J. thurifera*.

Population	SP_1	SP_2	SP_3	FR_1	FR_2	MO_1	MO_2	MO_3	MO_4	AL_1	AL_2
SP_1	–										
SP_2	0.011^**^	–									
SP_3	0.006^NS^	0.005^NS^	–								
FR_1	0.007^NS^	0.009^**^	0.003^NS^	–							
FR_2	0.032^*^	0.018^**^	0.011^**^	0.024^*^	–						
MO_1	0.012^**^	0.012^**^	0.003^NS^	0.013^**^	0.015^**^	–					
MO_2	0.023^*^	0.027^*^	0.013^**^	0.029^*^	0.033^*^	0.009^**^	–				
MO_3	0.009^**^	0.020^*^	0.006^NS^	0.020^*^	0.028^*^	0.002^NS^	0.005^NS^	–			
MO_4	0.015^**^	0.017^*^	0.007^NS^	0.016^*^	0.018^**^	0.003^NS^	0.005^NS^	0.006^NS^	–		
AL_1	0.035^*^	0.040^*^	0.032^*^	0.038^*^	0.059^*^	0.031^*^	0.034^*^	0.035^*^	0.032^*^	–	
AL_2	0.025^*^	0.024^*^	0.017^*^	0.019^*^	0.045^*^	0.021^*^	0.017^**^	0.025^*^	0.018^*^	0.020^**^	–

Level of significance at *P ≤ 0.001; **P ≤ 0.05; NS – non-significant.

### Geographic pattern of differentiation

AMOVA-based *k*-means clustering analysis identified *k* = 4 as the most likely number of clusters, and the pseudo-*F* statistic (Table 1 and Table 3) indicated clear genetic discontinuity among separate geographic regions. The European, Moroccan and Algerian populations formed three discrete groups. Substructure was detected within the European population; specifically, one Corsican population (FR_2) was detached from the Spanish populations. According to the BIC, populations were assigned to nine distinct groups (*k* = 9) with no clear geographic pattern (Table 1 and Table 3). This alternative partitioning indicated further substructure of populations within the main regions of the species’ range. Most populations were placed in separate clusters; only the SP_3 and FR_1 populations and two Moroccan populations (MO_1 and MO_3) shared the same clusters (cluster I and II, respectively).

**Table 3:** Summary of AMOVA-based K-means clustering conducted in GenoDive.

***k***	**SSD(T)**	**SSD(AC)**	**SSD(WC)**	**pseudo-*F***	**BIC**	**Rho**
2	3121.694	57.520	3064.174	2.479	63.552	0.021
3	3121.694	104.915	3016.779	2.600	63.119	0.034
4	3121.694	141.475	2980.219	2.644^*^	62.692	0.041
5	3121.694	163.935	2957.759	2.401	62.908	0.045
6	3121.694	185.111	2936.584	2.279	62.758	0.045
7	3121.694	204.101	2917.594	2.186	62.227	0.044
8	3121.694	222.991	2898.703	2.204	60.647	0.046
9	3121.694	239.011	2882.683	2.186	57.971^**^	0.047

The optimal number of clusters (*k*) indicated with two methods.

**Figure 2:**
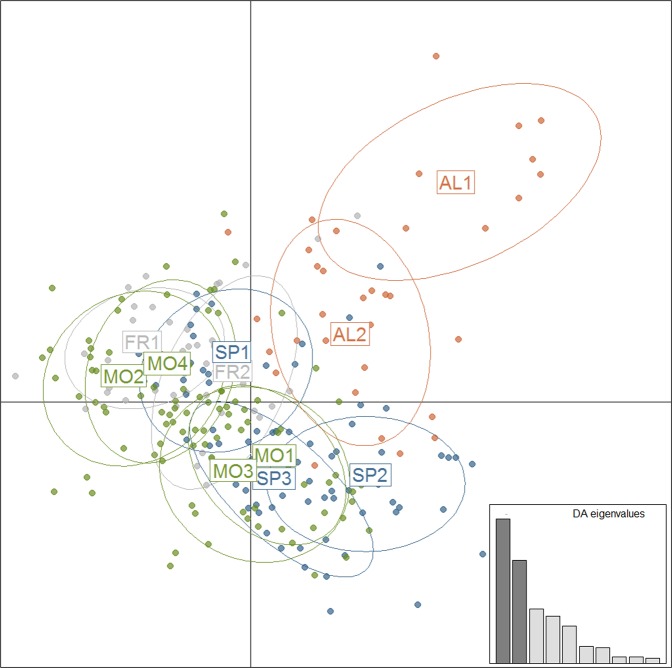
Ordination plot for the first two principal component axes resulting from a discriminant analysis of principal components (DAPC) for each individual, ellipses indicate their assignment to the genetic clusters inferred. The low-right graph indicates the variance explained by the principal component axes used for DAPC (dark grey).

DAPC identified the highest support for *k* = 4 based on the BIC and confirmed the pattern of differentiation revealed by the AMOVA-based K-means analysis. A clear geographic pattern of differentiation with moderate sharing of gene pools among inferred clusters was detected (Fig. 1A). It was also confirmed at the individual level on the ordination plot of the first two principal components out of 24 included (Fig. 2). Accordingly, all populations from the European part of the species’ range (SP and FR) were mostly assigned to clusters I and III with comparable average membership coefficients (44% and 37%, respectively). DAPC indicated further subdivision of the African populations into two groups with limited sharing of gene pools. The Moroccan populations were grouped mostly into clusters III and IV (average of 26% and 63%, respectively), whereas the Algerian population was put into cluster II (average 65%). Within the latter group, the most distinct population was Chelia (AL_1), which consisted of individuals showing limited genetic affinities to different clusters as compared with the other populations (cluster II, 83%).

According to STRUCTURE, the best K describing spatial genetic structure in analysed populations of *J. thurifera* was K = 6 (Fig. 1B,C). This result shows mostly the distinctiveness of the Algerian populations from the European and Moroccan stands. The Algerian populations formed rather homogenous group and were mostly assigned to cluster II with moderate gene exchange with other inferred clusters. All remaining populations were rather intermixed, but some general pattern could be inferred. The Moroccan populations were grouped mostly into clusters III and IV. Populations from the European part of the species’ range showed high gene admixture, but the Spanish populations were grouped mostly into clusters I and V and the Corsican population into clusters I and IV (Fig. 1B).

According to the Barrier analysis, three strongly supported genetic boundaries were identified among *J. thurifera* populations (Fig. 1A). Again, Algerian populations were indicated to be of a distinct character. They were clearly separated from the Spanish and Corsican (100% bootstrap value) and Moroccan (85% bootstrap value) populations. Additionally, barrier analysis also divided the two Algerian populations (98% bootstrap value) that were studied.

AMOVA showed that most of the genetic variation (94%) occurred within populations, while the variation attributable to geographic regions and genetic clusters defined by the clustering analysis accounted for 3.6% and 4.5% of the total variation, respectively (Table 4). A Mantel test revealed a significant positive correlation between the genetic and geographic distances (*r* = 0.334, P = 0.005).

**Table 4:** Analysis of molecular variance (AMOVA) estimated among geographic regions (Europe, Morocco and Algeria) and clusters revealed by clustering analysis conducted in GenoDive (*k* = 4).

Source of Variation	SSD	d.f.	MS	%Var	F-value	P-value
**Among regions**						
Within population	2797.118	235	11.903	0.938	0.062	–
Among population nested in regions	161.158	8	20.145	0.026	0.027	0.000
Among regions	99.813	2	49.907	0.036	0.036	0.000
**Among clusters (*k* = 4)**						
Within population	2797.118	235	11.903	0.938	0.062	–
Among population nested in clusters	123.257	7	17.608	0.017	0.018	0.001
Among clusters	137.715	3	45.905	0.045	0.045	0.000

Abbreviation: SSD - sum of squares deviations, df - degree of freedom, MS - mean squares, %var - percentage of total variation, P-value is based on 9,999 permutations.

### Modelling of species distributions

The MaxEnt model accuracy for present predictions of the *J. thurifera* distribution for WorldClim, expressed by the AUC, reached 0.974 for the combined European and African stands, 0.983 for European-only stands and 0.990 for African-only stands, indicating high model performance. Similarly high accuracy was obtained with the CHELSA dataset—0.972 for both African and European records, 0.983 for European-only records and 0.990 for African-only records. The most important factors limiting the current species range modelled jointly for the European and African datasets using WorldClim were the mean temperature of the coldest quarter (BIO11 – relative contribution: 28.6%), minimum temperature of the coldest month (BIO6 – relative contribution: 22.8%), mean temperature of the driest quarter (BIO9 – relative contribution: 11.4%) and precipitation of the warmest quarter (BIO18 – relative contribution: 10.2%) (Supplementary Table S3). The set of factors used to model the current distribution of the African subspecies was different only in terms of the number of important bioclimatic variables and their input (Supplementary Table S3). Nevertheless, BIO11 remained the most important (31.7%), the second critical variable was BIO18 (22.8%), and the remaining factors were of marginal influence. For the European range, some differences also appeared with respect to the strength of the variables. Contrary to the results of the joint analysis, the most important bioclimatic variable was BIO6 (24.0%), while BIO11 was the second most important (17.1%). In reference to the two remaining variables, BIO9 was less important (7.7%), and BIO18 made a relative contribution comparable to that in the joint analysis (11.6%).

Estimations made with the CHELSA dataset indicated a different set of factors that had the highest contribution to the model (Supplementary Table S4). The major difference relates to BIO14 (precipitation of the driest month). It was defined as the second most important variable affecting distribution of the African populations (28.7%), while WorldClim computations marginalized it (Supplementary Table S3). This bioclimatic variable had a very limited impact on the European distribution (3.1%), according to CHELSA. BIO6 remained significant, and its relative contribution was comparable in joint analysis (21.7%) to modelling using WorldClim (22.8%). However, using CHELSA, it was definitely higher for African-only (38.6%) as compared to European-only (10.3%) in comparison to WorldClim-based estimations (Supplementary Table S3). Contrary to WorldClim, modelling with CHELSA indicated that BIO1 (annual mean temperature) was also significant for African range (13.9% of contribution), while it was defined as being marginal in the case of WorldClim-based analysis. BIO11, which was indicated as affecting the African range at most according to WorldClim-based analysis (31.7%), turned out to be important for European-only modelling (17.6%), and for African populations only at negligible level (1.7%). The same situation was observed with BIO18, which had a high relative contribution for European-only estimations (16.7%) and low for African-only estimations (2.9%).

**Figure 3:**
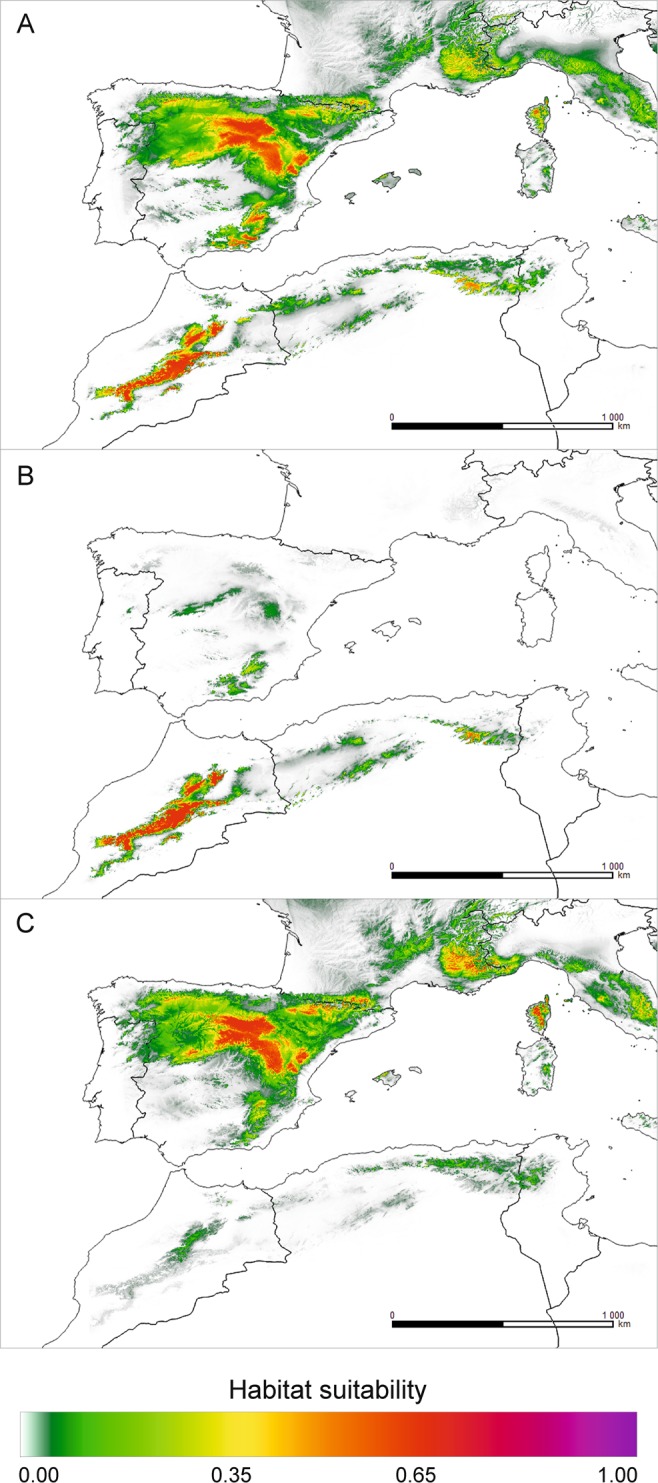
Theoretical current range of *J. thurifera*, estimated using MaxEnt based on raster data from the CHELSA database: (**A**) – European and African records; (**B**) – African-only records; (**C**) – European-only records. Map prepared with QGIS.

The theoretical contemporary range of *J. thurifera* modelled using both climatic datasets were very similar. Simulations of the range made jointly for the European and African data covered mainly the area of the Iberian Peninsula, with the most favourable conditions in the Central System and the Penibaetic System at the southern fringe of the Iberian Peninsula (Fig. 3A–C and, Supplementary Fig. S1, Fig. S2A–C). The same high suitability (65%) was also indicated for the southwestern Alps. In these areas, numerous records of species occurrence are available. Less favourable areas for *J. thurifera* were defined in the Pyrenees. Also, the Massif Central seems to possess habitats climatically suitable for the species, but no observations confirm its growth there. In reference to Africa, the High Atlas is the only location that may highly support the occurrence of *J. thurifera*, while in Algeria, only very limited areas in the Aurès Mountains offer suitable habitats. However, modelling the range separately for the African and European data revealed profound modification of expected distributions (Fig. 3B,C). Based on the European dataset, MaxEnt supported observed locations of the species in the Iberian Peninsula and in the Alps, but the African range was considered marginal (Supplementary Fig. S2). In contrast, modelling the distribution using African occurrences strongly supported the observed species occurrence in the High Atlas, and the suitability in the Aurès Mountains was much lower.

**Figure 4:**
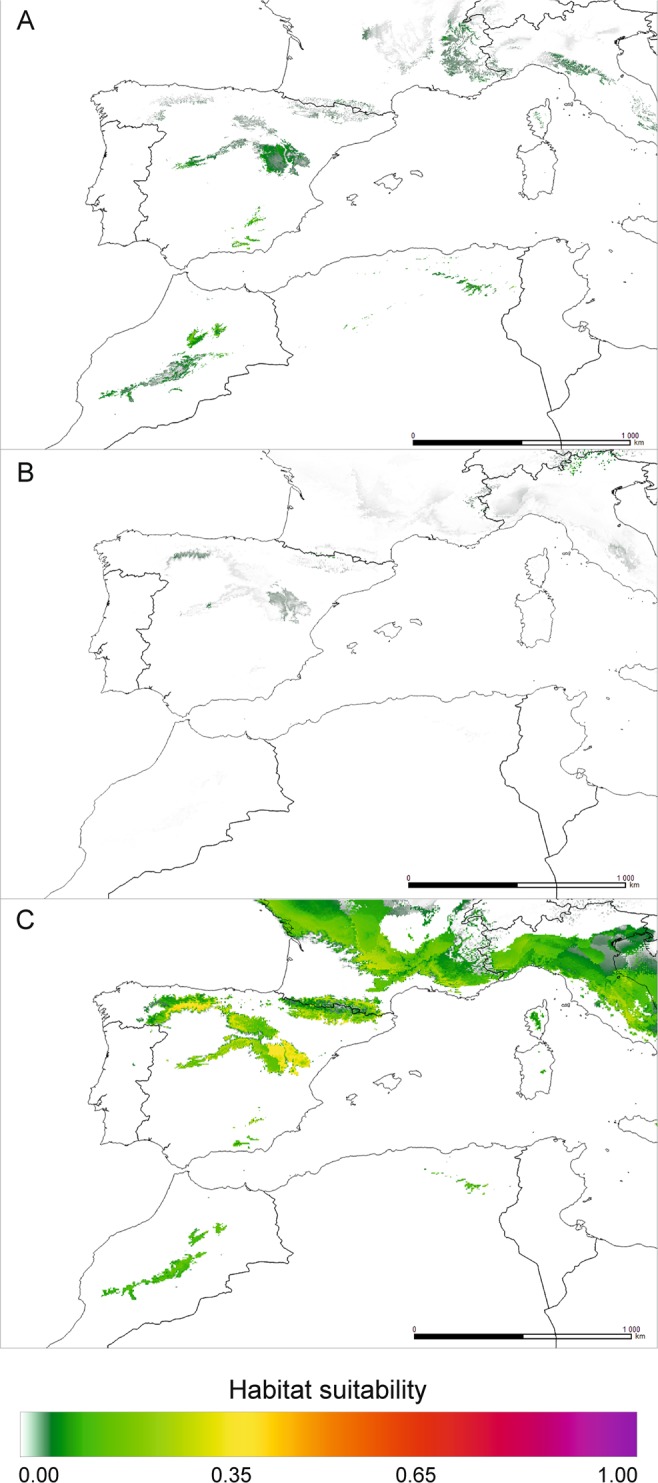
Theoretical range of *J. thurifera* in the past, estimated using MaxEnt based on raster data from WorldClim database: (**A**) – Eemian (*c*. 120–140 ka BP); (**B**) – LGM (*c*. 22,000 years ago), all records; (**C**) – LGM, European-only records. Map prepared with QGIS.

**Figure 5:**
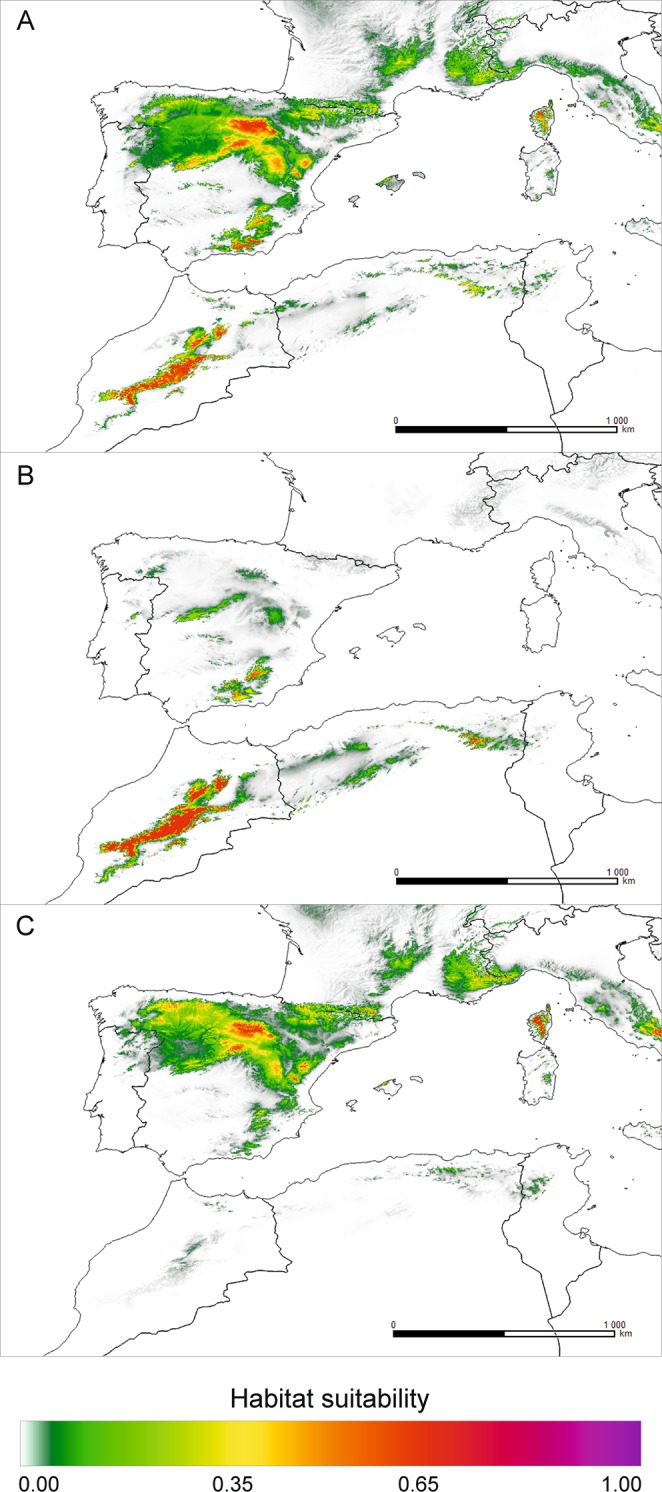
Theoretical range of *J. thurifera* in the future, estimated using MaxEnt based on raster data from the CHELSA database (year 2070): (**A**) – European and African records; (**B**) – African-only records; (**C**) – European-only records. The RCP 2.6 scenario of climate change was used for the CCSM4 model. Map prepared with QGIS.

Modelling the last interglacial distribution (Eemian) jointly for the European and African datasets (WorldClim) suggested a profound reduction of occurrence for *J. thurifera* (Fig. 4A). Only current core range areas in the Iberian Peninsula, the Alps and the High Atlas and Anti-Atlas were indicated as climatically suitable but with very low support (*c*. 5%). Analysis of the LGM based on WorldClim using all occurrence records (Fig. 4B) and that using only the African data (data not shown) failed to identify any suitable locations for *J. thurifera* during this period. Similarly, attempts to reconstruct the species’ range during the LGM using the CHELSA dataset gave no results for any of the combinations used (data not shown). The LGM distribution outlined based on the European-only data retrieved from WorldClim was vestigial to the current one (Fig. 4C). Suitable conditions may have existed in the Iberian Peninsula, probably in the Alps and to some extent in the Anti-Atlas and High Atlas. Generally, the species was likely severely limited (Fig. 4C). However, a dramatic reduction in the species distribution is projected under future climatic conditions based on WorldClim data (Supplementary material Fig. S3). Except for limited areas in the High Atlas, the species practically disappears. Projections made using climatic records from CHELSA are more optimistic (Fig. 5A–C). It predicts some reduction of the range when the datasets for both subspecies are considered. The niche modelling based on the Africa-only dataset indicated that, for African subspecies, the areas of the Penibaetic System in southern Spain may potentially deliver climatically suitable habitats in the future (Fig. 5B; suitability> 65%).

**Figure 6:**
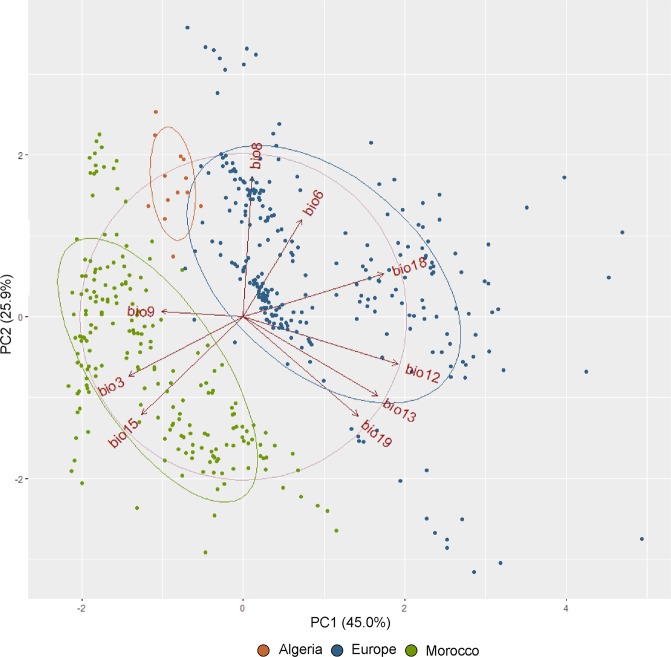
Influence of 19 bioclimatic variables on the current distribution of *J. thurifera* according to principal component analysis (PCA) based on 280 species occurrence data (acronyms of bioclimatic variables as in Table S3).

PCA analysis of bioclimatic dataset gained from CHELSA supported the ecological differentiation among European and African populations of *J. thurifera*. Accordingly, all species occurrence records were divided into two main groups according to the first two principal components (PC1 and PC2), which explained 70.9% of the total variation (Fig. 6). The component PC1 (45% of the total variation) was negatively correlated with isothermality (BIO3) and precipitation seasonality (BIO15) and positively correlated with variables that refer to precipitation (BIO12, BIO13, BIO18 and BIO19). The temperature variability (BIO6 and BIO8) and precipitation variables (BIO13, BIO15 and BIO19) contributed mainly to the PC2 component (25.9%) (Fig. 6; Supplementary material Table S5). However, in terms of climatic preferences, it seems that Algerian populations are more similar to European stands.

## Discussion

### North African phylogeographic congruencies include *J. thurifera* subsp. *africana*

The most striking result in our study was the recurrent genetic distinctiveness of the Algerian juniper populations from the Moroccan stands, as revealed by different analysis used in this study (Figs. 1 and 2). The pairwise F_st_ comparisons supported this distinct genetic character of the Algerian stands in reference to Spanish and Moroccan populations, with the latter two being more related to each other. General differentiation between European and African populations of *J. thurifera* (Fig. 1), was comparable to that obtained by Teixteira *et al*.^34^ but much lower than that revealed by Terrab *et al*.^37^ (3.6% here vs. 21.44% in Terrab *et al*.^37^). This difference probably reflects the different types of markers used in the latter study, particularly the dominant nature of AFLP markers. However, we observed higher differentiation (4.5%) the four genetic clusters detected (Fig. 1A), which points to a significant geographically driven pattern of differentiation in *J. thurifera*. The influence of geographic location was also supported by significant IBD. This result is certainly an effect of the spatial structure of the species’ natural range, being divided into a few isolated distributional domains in the complex mountainous landscape that currently limits gene flow and likely has hampered it in the past (Fig. 1A).

Our results on Moroccan-Algerian genetic split in *J. thurifera* subsp. *africana* are consistent with a few other intra-species diversification profiles noted among western Mediterranean plant and animal species that indicate shared spatial genetic structure^5,7,8^. The question of whether the genetic split in *J. thurifera* subsp. *africana* exemplifies the vicariance model or reflects separate migration pathways to eastern and western North Africa remains unanswered. In other words, is the Moroccan-Algerian divergence a secondary or primary phenomenon in the evolutionary history of *J. thurifera* in Africa?

According Jimenez *et al*.^20^, the colonization of Africa by the ancestor of modern *J. thurifera* might have taken place from the Iberian Peninsula *via* the Strait of Gibraltar. However, this conclusion was based solely on investigations of the Spanish and Moroccan stands. The earliest diversification time estimated for *J. thurifera* by Mao *et al*.^74^ was in the Oligocene/Miocene period (31.7–16.4 Ma). This matches the prominent geological rearrangements in the region that left detectable signals in the genetic structure of some species (*e.g*., *Quercus suber*^75^). The most recent estimates date to the Miocene/Pliocene (5.9–3.7 Ma), which is also acknowledged as a period of high diversification in the Mediterranean triggered by climatic changes^76^. According to geological reconstructions^77^, in both of these periods, especially in the Miocene, independent colonization of the African continent from the western and eastern directions was feasible. The largest number of private alleles that we recorded in Spanish populations (Table 1) confirms their long-term persistence and thus probably the general ancestral nature of the European gene pool in reference to the African one. However, this pattern does not preclude the independent two-directional colonization of Africa from Europe.

In contrast to our results, in the study of Terrab *et al*.^37^, closer genetic relationships of a single Algeria population to European ones were inferred, especially in reference to the Iberian range (Fig. 1 in Terrab *et al*.^36^). The two Algerian populations studied here clearly separated from the populations from the other regions, although they showed very limited admixture of the Spanish and Moroccan gene pools (Fig. 1). This genetic overlap might be interpreted as evidence of a common origin^20^ or ancient gene flow. Terrab *et al*.^36^ argued that long-distance dispersal events were the causal factors of the closer clustering of the single Algerian population to European ones. Inspection of a single herbarium individual from Algeria suggests high morphological similarity to European *J. thurifera* subsp. *thurifera*^35^. Although probable, this issue definitely requires more comprehensive morphometric analysis, including more populations and individuals from the Algerian range. However, the influence of the European gene pool needs to be considered in the case of the Algerian populations of *J. thurifera*. In fact, genetic connections across the Mediterranean Sea between North Africa and the central Mediterranean (Europe) have been demonstrated previously for *P. pinaster*^5^, *Laurus nobilis*^78^, *Carex panormitana*^79^ and *J. phoenicea*^80^, which suggests the complex evolutionary history of plant species in this part of the Mediterranean. Even more puzzling is the genetic affinity of Algerian and Tunisian populations of *A. glutinosa* to a single Scottish stand and not to geographically much closer Moroccan populations^8^. In terms of such unexpected genetic proximity, the authors concluded independent (including temporal) colonization of the western and eastern parts of North Africa by ancestral populations of *A. glutinosa*. Accordingly, Algerian and Tunisian populations are more recent colonizers, while Moroccan populations are defined as relicts. The diversification scenario proposed for the North African firs also involves two temporally and spatially independent colonization routes from Europe to North Africa^81^. The first one, depicted for the ancestor of *Abies pinsapo* and *A. maroccana*, would have run from the Iberian Peninsula and might have been related to the evolution of the Betic-Rif orogenic belt since the Miocene^82^. The second route dated to the Pliocene-Pleistocene would have involved colonization from the Apennine Peninsula and subsequent vicariance, resulting in the evolution of *A. numidica*^81^. Ultimately, the problem of colonization routes and their temporal aspects in the evolutionary history of *J. thurifera* subsp. *africana* remains unsolved in this study since solving it would require molecular dating and better sampling of the Algerian range. However, there is still ample evidence to consider the bidirectional colonization of North Africa, which is a convincing argument explaining the west-east genetic differentiation of *J. thurifera* subsp. *africana* and other plant and animal species in the region.

### Pleistocene/Holocene changes in species ranges: possible adaptation-driven divergence?

The impact of the Pleistocene glaciations, particularly the last glacial period, on the genetic patterns in North Africa is far less recognized than that on the European part of the Mediterranean, but it is invoked as a factor inducing patterns of endemism and differentiation^29,76,83^. Our species ecological niche modelling revealed interesting but very puzzling results. Surprisingly, projections made for the periods spanning the time from the Eemian (*c*. 120 ka BP) to the current time clearly suggest that the widest theoretical species occurrence is the one under current climatic conditions (Fig. 3A–C and, Supplementary Fig. S1, Fig. S2A–C). Meanwhile, it is commonly assumed that the rise of Holocene temperatures was the factor responsible for the reduction and fragmentation of the ranges of many *Juniperus* species occurring in the Mediterranean, and the environmental conditions during the glacial period promoted range expansion^20,34,44^. Another unexpected result from our projections concerns the mutually exclusive distribution patterns obtained when using the African-only or European-only dataset on species locations that may indicate climate-based adaptive divergence (Fig. 4B,C; Supplementary Fig. S2A–C).

Climatic conditions during the last glacial period led to the formation of glaciers in North African mountains, especially in the High Atlas^84^, while lower precipitation did not create suitable conditions for extended glacier formation in the Aurès Mountains^85^. Due to insufficient snow accumulation, relatively high annual temperatures and low precipitation at the highest points, glaciers are currently absent from the Atlas Mountains^86^. Consequently, the formation of glaciers in the High Atlas during the last glacial period would have required either (1) a significant decrease in the annual temperatures (by *c*. 7–8 °C) and the current level of precipitation or (2) more precipitation than currently observed and a smaller decrease in the mean annual temperatures^86^. *J. thurifera* subsp. *africana* prefers warm and dry habitats and exhibits strong continentalism; it is limited by the influence of the Atlantic climate^19^. Hence, irrespective of the climatic reconstruction supporting glacier formations in the High Atlas, the conditions for occurrence of *J. thurifera* subsp. *africana* were inadequate in North Africa. Consequently, MaxEnt failed to identify any climatically suitable conditions for the persistence of this taxon during the LGM in Africa based on current African-only locations using both climatic datasets (Fig. 4). However, the joint analysis of the European and African records made on the WorldClim dataset indicated some limited areas of species persistence in the Iberian Peninsula and West Alps but with very low suitability (*c*. 5%) (Fig. 4B). When using only the European records, in addition to the Iberian mountains and the Alps, optimal conditions for the species also appeared in the High Atlas and Aurès Mountains which could serve as refugia (Fig. 4C). Additional regions, such as the Pyrenees and Corsica, from which *J. thurifera* is reported today, were also indicated as suitable for refugia (Fig. 4C). Currently, this species occurs in Europe in areas with a total annual precipitation of at least 200 mm and a mean annual temperature of 8–10 °C^87^. Previous estimates of the potential distribution of several Mediterranean tree species from the Iberian Peninsula showed that the LGM range of *J. thurifera* was greatly limited and matched the one we modelled in our work^88^. It seems that, generally, climatic conditions for *J. thurifera* during the last glacial period were less suitable than the current theoretical ones, and its distribution expanded in the Holocene^82^. The factors affecting species occurrence in Europe during the LGM were related to temperature. The most significant for species occurrence were BIO6 (minimum temperature of the coldest month; 24.8%) and BIO11 (mean temperature of the coldest quarter; 18.2%). Current populations of African subspecies adapted to dry and hot climates could not be present during the LGM likely because of too low temperatures. The most important climatic factor affecting the distribution of *J. thurifera* subsp. *africana* (in fact a lack of its occurrence at that time) was BIO11 (31.2%). However, on the second position BIO18 (precipitation of the warmest quarter) was ranked (25.0%), which may suggest that it was also too arid during the LGM for current African taxa. According to Cheddadi *et al*.^83^, the mean annual temperatures in North Africa during the glacial period could have even been 15 °C lower than today, and the mean annual precipitation did not exceed 300 mm. In this way, our findings contradict previous assumptions made by some authors that the last cold period favoured a wider occurrence of this species^20,37^. Additionally, there is a compelling similarity between the Eemian (Fig. 4A,C. 120–140 ka BP) and glacial theoretical distribution (Fig. 4C) that suggests continuation of its occurrence in the core range in the Central System in the Iberian Peninsula, the West Alps, the High Atlas and the Aurès Mountains.

Given the mutually exclusive ranges projected with only African or only European records (Fig. 3), we hypothesize that climate-induced adaptation was one of the possible driving factors of the Ibero-African genetic differentiation recurrently revealed for *J. thurifera*, including in this work. According to the proposed scenario, spatial isolation in refugia defined with MaxEnt in Central System in Iberian Peninsula and Atlas Mountains in Africa (Fig. 4C) and the selective forces related to climatic changes in North Africa allowed genetic differentiation to arise and then be reinforced by genetic drift due to limited gene flow among different domains of the species distribution. First, MaxEnt models suggest that today’s European and African lineages are characterized by differential climatic requirements (Fig. 6, Supplementary Table S3 and Table S4). The most important climatic variables differ, and it seems that the African linage evolved towards enhanced drought resistance, as it is adapted to a dry continental climate with hot summers and low winter temperatures^19^ (Fig. 6, Supplementary Table S4). Second, the lack of climatic conditions favourable for the current African lineage, since at least the last interglacial, suggests its more recent evolution. Actually, for some species, the Quaternary glacial cycles were shown to be significant drivers of adaptation-based genetic divergence^89–91^. For *J. thurifera*, the crucial factor was probably the climate changes in North Africa throughout the Holocene. Reconstruction of the vegetation changes in the Middle Atlas based on Lake Sidi Ali, located at an altitude of 2,080 m above sea level, revealed the first occurrence of Cupressaceae (and so *Juniperus*) in the early Holocene and its expansion in the mid-Holocene^92^, which was cooler than the early Holocene. However, the general aridification trend in North Africa throughout the Holocene prevailed, and *Juniperus* withdrew at the expense of *Cedrus* only in more humid periods^92^. Our hypothesis of climate-driven differentiation between the European and African lineages is also somewhat supported at the morphological level. The biometric analysis revealed generally smaller dimensions of structures (cones and scales) and a smaller number of seeds per cone in the Moroccan populations as compared to in the European ones, which might be ascribed to adaptation to the severe arid climate of the Atlas Mountains^33^. However, this issue requires deeper investigations involving searching for genomic signals of adaptation^91^.

### Concluding remarks and perspectives

In this work, we demonstrated that Algerian populations of *J. thurifera* are a genetically distinct lineage (Figs. 1 and 2) and could be classified as *J. thurifera* var. *aurasiaca*, as previously suggested^35^. This species is one more excellent example of the extremely high intra-species genetic diversity noted in the North African region, representing another hotspot of evolution within the Mediterranean region. Unfortunately, this high biodiversity is presumed to face great environmental challenges that may lead to the irreversible loss of many species and thus plant community depletion. Ultimately, such a loss will affect ecosystem functioning and human well-being. In the African range of *J. thurifera*, the species is currently subjected to strong human pressure, fragmentation and reproductive failure^16^. All of these effects are negatively reinforced by ongoing aridification of the region, which makes populations vulnerable to additional disturbances and thereby exposes them to extinction risk. There are well-known examples of species in North Africa that are already trapped in the extinction vortex, such as endemic *Cupressus* or *Abies* species^29,76^. The most pessimistic projections of the future species distribution confirmed the serious reduction and almost extinction of *J. thurifera* (Supplementary Fig. S3). The predicted climatic deterioration is expected to affect the species’ range not only in Africa but also in Europe, leaving only a few locations of probable occurrence. Currently, *J. thurifera* is a key component of the high mountain forest communities of arid and semi-arid areas in North Africa and the Iberian Peninsula. A definitely more optimistic scenario was obtained using the CHELSA dataset (Fig. 5). The difference between CHELSA-based and WorldClim-based projections likely stems from the more precise performance of the first one^69^. Particularly, the precipitation algorithm that works in CHELSA includes orographic predictors such as wind fields or valley exposition with a subsequent bias correction that makes it work better and more reliable in case of the mountainous landscapes. Consequently, our CHELSA-based computations also indicated a reduction of the species’ range but not in such a drastic scale as WorldClim-based computations. In this case, the European part of the range was predicted to be more vulnerable for future climate changes than the African part of the range (Fig. 5 and Supplementary Fig. S3). The core range of the species in the Iberian mountains with the highest suitability (65%) was reduced profoundly (Fig. 5C). For African-based modelling, MaxEnt holds approximately the same areas of distribution as current ones and even projected the appearance of the suitable locations in the most southern fringes of the Iberian Peninsula (Fig. 5B). This particular result yearns for attention, as it allows the idea of assisted migration to be considered in the future to enhance the adaptability of the European population of the species.

Using a combined approach in which genetic analysis was supplemented with ecological niche modelling, we denoted the areas of the current species distribution that have constituted long-term refugia for *J. thurifera* since at least the Eemian (Fig. 4). Therefore, these areas represent the evolutionary heritage of the species and should thus be prioritized in conservation programmes^93^. Refugia are areas that have facilitated species survival during past climatic and environmental crises. However, as shown by our modelling, the buffering capacity of these future refugial areas in Central System in Iberian Peninsula (Fig. 5C) can be limited in the case of *J. thurifera* under expected environmental transformation. If the current trend in climate change continues^40^, these areas may no longer be efficient in supporting the species’ survival. Therefore, the evolutionary value of the Algerian populations should be immediately acknowledged by including them in a protected areas network, because they are more isolated and spatially confined than Moroccan stands.

As a final remark, we need to stress that predictions of future changes in species distribution done in this work are solely based on the climatic variables. Although they set the environmental boundaries of the species’ current or possible performance, they ignore local adaptations and so species evolvability. Meanwhile including evolutionary potential may change the predictions and fates of species^94^. However, genetic adaptation that may help to mitigate the climate-induced species and population demographic crisis leading to local extinctions require high levels of genetic diversity stored in populations and maintaining genetic connectivity among them. The more crucial is to focus conservation activity on species such as the fragmented and isolated *J. thurifera* subsp. *africana* in Algeria.

## Supplementary information

Supplementary information.
