# Supplementary material for: Patterns of genetic diversity in North Africa: Moroccan-Algerian genetic split in Juniperus thurifera subsp. africana
Source: Sci Rep. 2020 Mar 16;10:4810. doi: 10.1038/s41598-020-61525-x (PMC7075976; doi:10.1038/s41598-020-61525-x)

Patterns of genetic diversity in North Africa: Moroccan-Algerian genetic split in *Juniperus thurifera* subsp. *africana*

Asma Taib [1], Abdelkader Morsli [1], Aleksandra Chojnacka [2], Łukasz Walas [2], Katarzyna Sękiewicz [2], Adam Boratyński [2], Àngel Romo [3], Monika Dering [*2,4]

[1] Laboratoire de Ressources Génétiques et Biotechnologies, Ecole Nationale Supérieure Agronomique, Avenue Pasteur, Hassan Badi, 16200 Algiers, El Harrach, Algeria

[2] Institute of Dendrology Polish Academy of Sciences, Parkowa 5, 62-035 Kórnik, Poland

[3] Botanical Institute of Spanish Research Council, Passeig del Migdia, s/n 08038 Barcelona, Spain

[4] Department of Forest Silviculture, Poznań University of Life Sciences, Wojska Polskiego 71A, 60-637, Poznań, Poland

[*]Correspondence and requests for materials should be addressed to M.D. (email: mdering@man.poznan.pl)

Supplementary Table S1. Polymorphism of species-specific nSSR loci used in analysis.

Locus	Primer sequences 5' - 3'	Repeated motif	A	A_e	H_s
JT_01	AATCCATCACATGCCATCTTT<br>CCCTCATAAGAATCAATGAGATCC	TA_6	30	6.273	0.851
JT_04	CCAAGGAATGATCTAACCTTTGAA<br>TGGGATGCATATCTTATCTTCCT	AGA_7	12	1.908	0.482
JT_30	AATCCCCTATCCTTGCCAGT<br>TCAACAATATCAGCAAGTAATGAGA	TCT_{10}	14	4.927	0.806
JT_33	GAGCTTCCTTTGTAGATTTTGGG<br>GTAAGAAGACACCACTCAGTCGAT	CT_{11}	44	10.154	0.912
JT_40	GGCCGCATGATCCATTACT<br>TCGTAACGTAATGACATGTATAGTGC	CA_{20}	27	5.115	0.814
JT_46	TGAGATCACCTACTTCCTAGTGGA<br>CCACCAAGGGCATAGAGTTC	AGG_7	17	4.158	0.768
		**Average**	**24 (144)**	**5.423**	**0.772**

Abbreviation: A - number of alleles observed; A_e - effective number of alleles; H_s - heterozygosity within populations; in brackets - average number of alleles observed

Supplementary Table S2. Locations of all points used in MaxEnt analysis.

Country	Region	Locality	Latitude	Longitude	Altitude	Year	Literature or observation
Algeria	Aurès Mts.	Chelia	35.300	6.620	1910	2013	Beghami Y. et al. 2013
Algeria	Aurès Mts.	Chelia	35.003	6.006	-	2018	Personal observation (Taib)
Algeria	Aurès Mts.	Jbel Ahmar Khaddou	35.367	6.417	1760	2008	Terrab et al. 2008
Algeria	Aurès Mts.	Oued Abdi	35.370	6.340	1800	2013	Beghami Y. et al. 2013
Algeria	Aurès Mts.	Oued Abdi	35.300	6.000	1412	2013	Beghami Y. et al. 2013
Algeria	Aurès Mts.	Oued Abdi	35.380	6.300	1300	2017	Bach et al. 2017
Algeria	Aurès Mts.	S'Gag	35.380	6.160	1900	2013	Beghami Y. et al. 2013
Algeria	Aurès Mts.	Tafrent	35.002	6.006		2018	Personal observation (Taib)
Algeria	Aurès Mts.	Tibhirine	35.310	6.400	1560	2013	Beghami Y. et al. 2013
Algeria	Aurès Mts.	T'kout	35.100	6.390	1370	2013	Beghami Y. et al. 2013
Algeria	Aurès Mts.	T'kout	35.160	6.390	1675	2013	Beghami Y. et al. 2013
Algeria	Aurès Mts.	T'kout	35.170	6.450	1450	2013	Beghami Y. et al. 2013
Algeria	Aurès Mts.	T'kout	35.050	6.600	1477	2013	Beghami Y. et al. 2013
Algeria	Aurès Mts.	T'kout	35.030	6.600	1553	2013	Beghami Y. et al. 2013
Algeria	Aurès Mts.	T'kout	35.250	6.500	1742	2013	Beghami Y. et al. 2013
France	Alpes-Maritimes	Isola	44.180	7.080	920	1983	Borel and Polidori 1983
France	Alpes-Maritimes	Isola	44.180	7.060	970	1983	Borel and Polidori 1983
France	Alpes-Maritimes	Isola	44.190	7.050	1050	1983	Borel and Polidori 1983
France	Alpes-Maritimes	Valabre	44.130	7.090	850	1983	Borel and Polidori 1983
France	Alpes-Maritimes	Valabre	44.130	7.090	1040	1983	Borel and Polidori 1983
France	Alpes-Maritimes	Valabre	44.140	7.090	1190	1983	Borel and Polidori 1983
France	Alpes-Maritimes	Valabre	44.140	7.110	1120	1983	Borel and Polidori 1983
France	Alpes-Maritimes	Valabre	44.130	7.100	850	1983	Borel and Polidori 1983
France	Alpes-Maritimes	Valabre	44.150	7.100	870	1983	Borel and Polidori 1983
France	Alpes-Maritimes	Valabre	44.160	7.090	830	1983	Borel and Polidori 1983
France	Alpes-Maritimes	vallon de Adousses	44.110	7.100	850	1983	Borel and Polidori 1983
France	Alpes-Maritimes	vallon de Longon	44.120	7.090	780	1983	Borel and Polidori 1983
France	Alpes-Maritimes	vallon de Longon	44.120	7.070	1250	1983	Borel and Polidori 1983

France	Alpes-Maritimes	vallon de Longon	44.120	7.070	1130	1983	Borel and Polidori 1983
France	Alpes-Maritimes	vallon de Molières	44.130	7.130	1000	1983	Borel and Polidori 1983
France	Alpes-Maritimes	vallon de Molières	44.130	7.120	1300	1983	Borel and Polidori 1983
France	Alpes-Maritimes	vallon de Molières	44.130	7.120	1150	1983	Borel and Polidori 1983
France	Alpes-Maritimes	vallon de Molières	44.130	7.110	980	1983	Borel and Polidori 1983
France	Alpes-Maritimes	vallon de Molières	44.140	7.140	1160	1983	Borel and Polidori 1983
France	Alpes-Maritimes	vallon de Molières	44.140	7.150	1410	1983	Borel and Polidori 1983
France	Alpes-Maritimes	vallon de Molières	44.140	7.140	1280	1983	Borel and Polidori 1983
France	Alpes-Maritimes	vallon de Molières	44.150	7.150	1380	1983	Borel and Polidori 1983
France	Alpes-Maritimes	vallon de Molières	44.140	7.150	1320	1983	Borel and Polidori 1983
France	Alpes-de-Haute-Provance	Le Cheinet	44.110	6.570	1180	2013	Legland et al. 2013
France	Alpes-de-Haute-Provance	Les Vignes	44.270	6.260	1150	2013	Legland et al. 2013
France	Alpes-de-Haute-Provance	Roche des Prises	44.370	6.070	950	2013	Legland et al. 2013
France	Alpes-de-Haute-Provance	Roche Juan	44.400	6.520	1220	2013	Legland et al. 2013
France	Alpes-de-Haute-Provance	Rocher de la Meichira	44.220	6.440	1050	2013	Legland et al. 2013
France	Alpes-Maritimes	Rocher du Susarme	44.090	7.590	1080	2013	Legland et al. 2013
France	Alpes-Maritimes	Saint-Sauveur-sur-Tinée	44.100	7.120	870	2013	Legland et al. 2013
France	Alpes-Maritimes	Vallerase	44.010	7.460	1050	2013	Legland et al. 2013
France	Corsica	Corsica	42.350	9.050	748	2013	Romo et al. 2013
France	Corsica	Foret de Melaja	42.510	8.950	1200	1994	Gamisans 1994
France	Corsica	Monte Agutu	42.340	9.050	880	1994	Gamisans 1994
France	Corsica	Niolu	42.533	9.050	-	2008	Terrab et al. 2008
France	Corsica	Scala Santa Regina	42.370	9.090	490	1994	Gamisans 1994
France	Corsica	Scala Santa Regina	42.370	9.080	540	1994	Gamisans 1994
France	Corsica	Scala Santa Regina	42.360	9.080	800	1994	Gamisans 1994
France	Corsica	Vallée d'Asco	42.430	9.030	750	1994	Gamisans 1994
France	Corsica	Vallée d'Asco	42.410	9.030	1050	1994	Gamisans 1994
France	Corsica	Vallée d'Asco	42.410	9.030	1200	1994	Gamisans 1994
France	Corsica	Vallée d'Asco	42.440	9.040	1000	1994	Gamisans 1994
France	Corsica	Vallon de Rudda	42.390	9.040	1350	1994	Gamisans 1994
France	Corsica	Vallon de Rudda	42.380	9.030	1200	1994	Gamisans 1994
France	Corsica	Vallon de Rudda	42.380	9.040	1350	1994	Gamisans 1994

France	Corsica	Vallon de Rudda	42.380	9.020	1250	1994	Gamisans 1994
France	Corsica	Vallon Pruniccia (Tribbiatoghia)	42.340	9.050	700	1994	Gamisans 1994
France	Corsica	Vallon Pruniccia (Tribbiatoghia)	42.340	9.060	1000	1994	Gamisans 1994
France	Corsica	Vallon Pruniccia (Tribbiatoghia)	42.340	9.060	900	1994	Gamisans 1994
France	Corsica	Vallon Pruniccia (Tribbiatoghia)	42.340	9.060	800	1994	Gamisans 1994
France	Corsica	Vallon Pruniccia (Tribbiatoghia)	42.340	9.070	1100	1994	Gamisans 1994
France	Drôme	Laval	45.000	5.340	400	2013	Legland et al. 2013
France	Drôme	Les Aiguilles	44.440	5.450	880	2013	Legland et al. 2013
France	Drôme	Les Jacots	45.000	5.250	820	2013	Legland et al. 2013
France	Drôme	Mornans	44.630	5.080	550	2013	Legland et al. 2013
France	Drôme	Reychas	44.680	5.570	1050	2013	Legland et al. 2013
France	Drôme	Rocher de Sainte-Croix	43.760	6.150	500	2013	Legland et al. 2013
France	Haute Garonne	Marignac Montagne de Rié	42.890	-0.660	1130	2013	Romo et al. 2013
France	Hautes-Alpes	St. Crépin	44.710	6.610	1160	2013	Romo et al. 2013
France	Hautes-Alpes	Chanousse	44.360	5.660	750	2013	Legland et al. 2013
France	Hautes-Alpes	La Roche-des-Arnouds	44.550	5.900	-	2008	Terrab et al. 2008
France	Hautes-Alpes	Montmaur	44.600	5.860	1150	2013	Legland et al. 2013
France	Hautes-Alpes	Montmaur	44.560	5.910	990	2013	Legland et al. 2013
France	Hautes-Alpes	Pierre Feu	44.470	6.220	1050	2013	Legland et al. 2013
France	Hautes-Alpes	Revuaire	44.390	5.780	1100	2013	Legland et al. 2013
France	Hautes-Alpes	Sain-André-de-Rosans	44.360	5.510	700	2013	Legland et al. 2013
France	Hautes-Alpes	Saint Crepin	44.700	6.600	-	2008	Terrab et al. 2008
France	Hautes-Alpes	Serre la Croix	44.580	5.740	1100	2013	Legland et al. 2013
France	Hautes-Alpes	Comboire	45.150	5.680	480	2013	Vela, Schäfer 2013
France	Hautes-Alpes	Grottes de Choranche	45.070	5.400	800	2013	Legland et al. 2013
France	Hautes-Alpes	Le Charpenet	45.260	5.690	640	2013	Legland et al. 2013
France	Hautes-Alpes	Roches de Comboire	45.150	5.680	480	2013	Legland et al. 2013
France	Hautes-Alpes	Saint-Clément pres d' Embrun	44.640	6.570	970	2013	Vela, Schäfer 2013
France	Hautes-Alpes	Saint-Égrève allant à Néron	45.230	5.700	850	2013	Vela, Schäfer 2013
France	Pyrenees	Haute-Garonne	43.783	0.100	-	2008	Terrab et al. 2008
France	Pyrenees	Quié de Lujat	42.800	1.660	750	2003	Gauquelin et al. 2003
France	Pyrenees	Quié de Lujat	42.810	1.660	1050	2003	Gauquelin et al. 2003

France	Pyrenees	Quié de Lujat	42.810	1.670	1450	2003	Gauquelin et al. 2003
France	Pyrenees	Quie de Vebre	42.780	1.720	910	2003	Gauquelin et al. 2003
France	Pyrenees	Saint Beat	42.910	0.680	840	2003	Gauquelin et al. 2003
France	Ryrenees	Quié de Lujat	42.790	1.670	1010	2013	Romo et al. 2013
France	Savoie	La Thuile	45.510	6.040	670	2013	Legland et al. 2013
Italy	Alpes-Maritimes	Valdieri	44.267	7.383	-	2008	Terrab et al. 2008
Morocco	Anti-Atlas	Jbel Sarhro			2712	1939	Emberger 1939
Morocco	Anti-Atlas	Jbel Sarhro	31.120	-5.720	2260	2008	Personal observation (Romo & Boratyński)
Morocco	Anti-Atlas	Jbel Sarhro	31.120	-5.650	2200	2011	Personal observation (Romo & Boratyński)
Morocco	Anti-Atlas	Jbel Sarhro	31.120	-5.640	2000-2100	1941	Emberger and Maire 1941
Morocco	Anti-Atlas	Jbel Sarhro	31.140	-5.720	2180	2008	Personal observation (Romo & Boratyński)
Morocco	Anti-Atlas	Jbel Sarhro	31.140	-5.700	2400	2008	Personal observation (Romo & Boratyński)
Morocco	Anti-Atlas	Jbel Sarhro	31.150	-5.900	2350	2008	Personal observation (Romo & Boratyński)
Morocco	Anti-Atlas	Jbel Sarhro	31.150	-5.840	2390	2008	Personal observation (Romo & Boratyński)
Morocco	Anti-Atlas	Jbel Sarhro	31.150	-5.840	2550	2003	Personal observation (Dobingard)
Morocco	Anti-Atlas	Jbel Sarhro	31.150	-5.820	2400	2008	Personal observation (Romo & Boratyński)
Morocco	Anti-Atlas	Jbel Sarhro	31.150	-5.810	2500	2008	Personal observation (Romo & Boratyński)
Morocco	Anti-Atlas	Jbel Sarhro	31.150	-5.660	2200	1994	Quézel, Barbero & Rivas Mart. 1994
Morocco	Anti-Atlas	Jbel Sarhro	31.150	-5.660	255 -2700	2003	Personal observation (Dobingard)
Morocco	Anti-Atlas	Jbel Sarhro	31.160	-5.880	2350	2008	Personal observation (Romo & Boratyński)
Morocco	Anti-Atlas	Jbel Sarhro	31.160	-5.820	2360	2008	Personal observation (Romo & Boratyński)
Morocco	Anti-Atlas	Jbel Sarhro	31.170	-5.870	2240	2008	Personal observation (Romo & Boratyński)
Morocco	Anti-Atlas	Jbel Sarhro	31.170	-5.850	2470	2008	Personal observation (Romo & Boratyński)
Morocco	Anti-Atlas	Jbel Sarhro	31.180	-5.820	2350	2008	Personal observation (Romo & Boratyński)
Morocco	Anti-Atlas	Jbel Siroua	30.700	-7.620	2700	2003	Personal observation (Dobingard)
Morocco	Anti-Atlas	Jbel Siroua	30.700	-7.620	2800-3200	2003	Personal observation (Dobingard)
Morocco	High Atlas	Azzadem Oussem	31.120	-7.960	2331	2014	Teixeira et al. 2014
Morocco	High Atlas	Matat	31.150	-7.960	2060	2014	Teixeira et al. 2014
Morocco	High Atlas	Oukaimeden	31.242	-7.817	2120	2014	Personal observation (Boratyński, Romo, Dering, Sękiewicz & Sękiewicz)
Morocco	High Atlas	Oukaimeden	31.200	-7.880	2600	2014	Teixeira et al. 2014
Morocco	High Atlas	Oukaimeden	31.200	-7.880	2540	2014	Teixeira et al. 2014

Morocco	High Atlas	Tizi Techt	31.170	-7.970	2033	2014	Teixeira et al. 2014
Morocco	High Atlas	Tizrag	31.220	-7.880	2700	2013	Sanguin et al. 2013; El Alauli el Fels et al. 2013
Morocco	High Atlas	Zaouïa-Ahansal	31.960	-6.120	1900	2013	Romo et al. 2013
Morocco	High Atlas	Acif Aït Maesan	31.130	-7.910	1950	1923	Personal observation (Litardiere)
Morocco	High Atlas	Adrar Agoumi	31.110	-7.960	2350	1988	Gauquelin & Dagnac 1988
Morocco	High Atlas	Agoundis et Zaout	30.970	-8.060	2400	2000	Ouhammou et al. 2000
Morocco	High Atlas	Ahansal	31.790	-6.080	2000	1981	Quezel and Barbero 1981
Morocco	High Atlas	Ahansal	31.810	6.100	2350	1999	El Alaoui et al. 1999
Morocco	High Atlas	Ait Bou Guemmez	31.650	-6.420	1850	1999	El Alaoui et al. 1999
Morocco	High Atlas	Ait Bou Guemmez	31.650	-6.360	2350	1988	Gauquelin & Dagnac 1988
Morocco	High Atlas	Ait Bou Guemmez	31.670	-6.430	2100	1988	Gauquelin & Dagnac 1988
Morocco	High Atlas	Aït Daoud	31.830	-5.470	2100	2001	Blali 2001
Morocco	High Atlas	Aït Messane (Mizane)	31.120	-7.890	2300	1926	Personal observation (Lindberg)
Morocco	High Atlas	Akka n'Tazzert	31.720	-5.300	2650	1957	Maire 1957
Morocco	High Atlas	Amismiz	31.070	-8.380	2600	1925	Personal observation (Maire)
Morocco	High Atlas	Azzadem	31.130	-7.960	2350	1999	El Alaoui et al. 1999
Morocco	High Atlas	Azzadem	31.130	-7.960	2000-2600	2002	Gauquelin et al. 2002
Morocco	High Atlas	Azzadem	31.140	-7.960	2250	1988	Gauquelin and Dagnac 1988
Morocco	High Atlas	Azzadem	31.160	-7.950	2300	2000	Ouhammou et al. 2000
Morocco	High Atlas	Ighighayene Valley	31.220	-7.850	2300	2002	Gauquelin et al. 2002
Morocco	High Atlas	Ighighayene Valley	31.220	-7.840	2400	2002	Gauquelin et al. 2002
Morocco	High Atlas	Ighighayene Valley	31.220	-7.840	2550	2002	Gauquelin et al. 2002
Morocco	High Atlas	Ighighayene Valley	31.230	-7.860	2050	2002	Gauquelin et al. 2002
Morocco	High Atlas	Ighighayene Valley	31.230	-7.850	2150	2002	Gauquelin et al. 2002
Morocco	High Atlas	Ilmil	31.180	-7.950	2250-2550	1989	Personal observation (Podlech)
Morocco	High Atlas	Jbel Aioui	31.800	-6.160	2500-2800	1981	Quezel and Barbero 1981
Morocco	High Atlas	Jbel Azourki	31.720	-6.350	2100	2004	Personal observation (Romo)
Morocco	High Atlas	Jbel Azourki	31.770	-6.280	2600	2004	Personal observation (Romo)
Morocco	High Atlas	Jbel Azourki	31.780	-6.240	2600	2005	Personal observation (Romo, Boratyński & Didukh)
Morocco	High Atlas	Jbel Azourki	31.790	-6.230	2400	1999	El Alaoui et al. 1999
Morocco	High Atlas	Jbel Azourki	31.790	-6.230	2480	2005	Personal observation (Romo, Boratyński & Didukh)
Morocco	High Atlas	Jbel Azourki	31.790	-6.230	1800-2450	2002	Gauquelin et al. 2002

Morocco	High Atlas	Jbel Azourki	31.800	-6.230	2400	2005	Personal observation (Romo, Boratyński & Didukh)
Morocco	High Atlas	Jbel Azourki	31.800	-6.230	2450	2005	Personal observation (Romo, Boratyński & Didukh)
Morocco	High Atlas	Jbel Azourki	31.800	-6.210	2370	2005	Personal observation (Romo, Boratyński & Didukh)
Morocco	High Atlas	Jbel Azourki	31.800	-6.210	2420	2005	Personal observation (Romo, Boratyński & Didukh)
Morocco	High Atlas	Jbel Azourki	31.810	-6.240	2480	2005	Personal observation (Romo, Boratyński & Didukh)
Morocco	High Atlas	Jbel Azourki	31.810	-6.240	2530	2005	Personal observation (Romo, Boratyński & Didukh)
Morocco	High Atlas	Jbel Azourki	31.810	-6.230	2330	2005	Personal observation (Romo, Boratyński & Didukh)
Morocco	High Atlas	Jbel Azourki	31.810	-6.220	2350	2005	Personal observation (Romo, Boratyński & Didukh)
Morocco	High Atlas	Jbel Azourki	31.810	-6.220	2450	2005	Personal observation (Romo, Boratyński & Didukh)
Morocco	High Atlas	Jbel Azourki	31.830	-6.250	2200	1981	Quezel and Barbero 1981
Morocco	High Atlas	Jbel Azourki	31.830	-6.220	2550	2005	Personal observation (Romo, Boratyński & Didukh)
Morocco	High Atlas	Jbel Azourki	31.840	-6.270	2100	1981	Quezel and Barbero 1981
Morocco	High Atlas	Jbel Azourki	31.850	-6.240	2300	2005	Personal observation (Romo, Boratyński & Didukh)
Morocco	High Atlas	Jbel Badiy	32.500	-6.660	2500	1988	Gauquelin & Dagnac 1988
Morocco	High Atlas	Jbel Ghat	31.530	-6.930	2000	1938	Emberger 1938
Morocco	High Atlas	Jbel Gourza	31.050	-8.240	2700	2000	Alifiqui 2000
Morocco	High Atlas	Jbel Imïdghäs	32.390	-5.200	2520	1938	Emberger 1938
Morocco	High Atlas	Jbel Imïdghäs	32.400	-5.210	2890	1939	Emberger 1939
Morocco	High Atlas	Jbel M'Goum	31.480	-6.440	-	1939	Emberger 1939
Morocco	High Atlas	Jbel Sloul	32.290	-5.360	1950	1987	Quézel, Barbero and Benabid 1987
Morocco	High Atlas	Jbel Sloul	32.290	-5.360	1950	1987	Quézel, Barbero and Benabid 1987
Morocco	High Atlas	Jbel Tighardine	31.150	-8.160	2200	2000	Alifriqui 2000
Morocco	High Atlas	Jbel Tirhordine	32.300	-5.320	2000	1987	Quézel, Barbero and Benabid 1987
Morocco	High Atlas	Jbel Tirhordine	32.300	-5.320	2000	1987	Quézel, Barbero and Benabid 1987
Morocco	High Atlas	Jbel Toubkal	31.090	-7.920	2605	1994	Benabid and Fennane 1994
Morocco	High Atlas	Jbel Toubkal	31.090	-7.920	2800	2000	Alifiqui 2000
Morocco	High Atlas	Jbel Toubkal	31.090	-7.780	2700	2000	Alifriqui 2000
Morocco	High Atlas	Jbel Toubkal	31.100	-7.950	2805	1994	Benabid and Fennane 1994
Morocco	High Atlas	Jbel Toubkal	31.110	-7.920	2250	2000	Alifiqui 2000
Morocco	High Atlas	N'Fiss	30.880	-8.430	2200-2500	2002	Gauquelin et al. 2002
Morocco	High Atlas	N'Fiss	30.890	-8.290	2350	1999	Al. Alaoui et al. 1999
Morocco	High Atlas	N'Fiss	30.890	-8.290	-	2000	Alifriqui 2000

Morocco	High Atlas	Oued d' Tessaout	31.400	-6.890	2000	2005	Personal observation (Romo, Boratyński & Didukh)
Morocco	High Atlas	Oued d' Tessaout	31.410	-6.900	2200	2005	Personal observation (Romo, Boratyński & Didukh)
Morocco	High Atlas	Oued d' Tessaout	31.420	-6.920	2400	2005	Personal observation (Romo, Boratyński & Didukh)
Morocco	High Atlas	Oued d' Tessaout	31.420	-6.910	2200	2005	Personal observation (Romo, Boratyński & Didukh)
Morocco	High Atlas	Oued d' Tessaout	31.420	-6.900	2200	2005	Personal observation (Romo, Boratyński & Didukh)
Morocco	High Atlas	Oued d' Tessaout	31.420	-6.890	2000	2005	Personal observation (Romo, Boratyński & Didukh)
Morocco	High Atlas	Oued d' Tessaout	31.430	-6.920	2340	2005	Personal observation (Romo, Boratyński & Didukh)
Morocco	High Atlas	Oued d' Tessaout	31.430	-6.920	2370	2005	Personal observation (Romo, Boratyński & Didukh)
Morocco	High Atlas	Oued d' Tessaout	31.430	-6.890	2000	2005	Personal observation (Romo, Boratyński & Didukh)
Morocco	High Atlas	Oukaimeden	31.200	-7.870	2500	1994	Personal observation (Jury et al.)
Morocco	High Atlas	Oukaimeden	31.210	-7.850	2600	2002	Personal observation (Romo & Boratyński)
Morocco	High Atlas	Oukaimeden	31.220	-7.850	2600	1985	Gauquelin 1985
Morocco	High Atlas	Oukaimeden	31.220	-7.850	2100-2600	2002	Gauquelin et al. 2002
Morocco	High Atlas	Oukaimeden	31.220	-7.850	-	1999	Gauquelin et al. 1999
Morocco	High Atlas	Oukaimeden	31.220	-7.850	-	2001	Jury 2001
Morocco	High Atlas	Oukaimeden	31.220	-7.850	-	1921	Maire 1921
Morocco	High Atlas	Oukaimeden	31.220	-7.850	-	1991	Ouhammou 1991
Morocco	High Atlas	Oukaimeden	31.220	-7.850	-	2000	Personal observation (Jury)
Morocco	High Atlas	Oukaimeden	31.230	-7.820	2400	1988	Gauquelin & Dagnac 1988
Morocco	High Atlas	Ourika-Zat	31.220	-7.600	2700	2000	Alifriqui 2000
Morocco	High Atlas	Tirrhist	32.100	-5.640	2200	1981	Quezel and Barbero 1981
Morocco	High Atlas	Tirrhist	32.160	-5.630	2000	1981	Quezel and Barbero 1981
Morocco	High Atlas	Tirrhist	32.170	-5.630	2100	1981	Quezel and Barbero 1981
Morocco	High Atlas	Tirrhist	32.170	-5.630	2150	1981	Quezel and Barbero 1981
Morocco	High Atlas	Tirrhist	32.180	-5.630	2100	1981	Quezel and Barbero 1981
Morocco	High Atlas	Tirrhist	32.180	-5.630	2150	1981	Quezel and Barbero 1981
Morocco	High Atlas	Tirrhist	32.180	-5.630	2200	1981	Quezel and Barbero 1981
Morocco	High Atlas	Tirrhist	32.210	-5.420	2100	1981	Quezel and Barbero 1981
Morocco	High Atlas	Tirrhist	32.220	-5.430	2000	1981	Quezel and Barbero 1981
Morocco	High Atlas	Tirrhist	32.230	-5.430	2000	1981	Quezel and Barbero 1981
Morocco	High Atlas	Tirrhist	32.240	-5.430	2000	1981	Quezel and Barbero 1981
Morocco	High Atlas	Tirrhist	32.260	-5.410	2850	1978	Peyre 1978

Morocco	High Atlas	Tizi n'Ilissi	31.830	-6.170	2400	1957	Maire 1957
Morocco	High Atlas	Tizi n'Ilissi	31.830	-6.170	2400	1988	Gauquelin & Dagnac 1988
Morocco	High Atlas	Tizi n'Ilissi	31.830	-6.170	2410	1981	Quezel and Barbero 1981
Morocco	High Atlas	Tizi n'Ilissi	31.830	-6.160	2250	1981	Quezel and Barbero 1981
Morocco	High Atlas	Tizi n'Ilissi	31.830	-6.160	2300	1981	Quezel and Barbero 1981
Morocco	High Atlas	Tizi n'Ilissi	31.830	-6.150	2100	1981	Quezel and Barbero 1981
Morocco	High Atlas	Tizi n'Ilissi	31.830	-6.150	2210	1981	Quezel and Barbero 1981
Morocco	High Atlas	Tizi n'Ilissi	31.830	-6.100	1650	1994	El Alaoui et al.. 1994
Morocco	High Atlas	Tizi n'Ilissi	31.830	-6.100	1650	1999	Gauquelin et al. 1999
Morocco	High Atlas	Tizi n'Ilissi	31.840	-6.170	2400	1981	Quezel and Barbero 1981
Morocco	High Atlas	Tizi n'Ilissi	31.840	-6.170	2450	1981	Quezel and Barbero 1981
Morocco	High Atlas	Tizi n'Ilissi	31.840	-6.170	2450	1988	Gauquelin & Dagnac 1988
Morocco	High Atlas	Tizi n'Mzic	31.110	-7.950	2300	1987	Maire 1957
Morocco	High Atlas	Tizi n'Mzic	31.110	-7.950	-	2001	Gómiz 2001
Morocco	High Atlas	Tizi n'Test	30.900	-8.300	1700	1952	Personal observation (Retz)
Morocco	High Atlas	Tizi n'Tichka	31.270	-7.360	1970	1981	Quezel and Barbero 1981
Morocco	High Atlas	Tizi n'Tichka	31.280	-7.380	2200	1981	Quézel and Barbero 1981
Morocco	High Atlas	Tizi n'Tichka	31.280	-7.380	2200	2002	Personal observation (AR, AB)
Morocco	High Atlas	Tizi n'Tichka	31.330	7.340	1850-2100	1981	Quezel and Barbero 1981
Morocco	High Atlas	Tizi n'Tichka	31.330	7.340	1850-2100	1981	Quezel and Barbero 1981
Morocco	High Atlas	Tizi n'Tichka	31.330	7.340	1850-2100	1981	Quezel and Barbero 1981
Morocco	High Atlas	Tizrag	31.300	-7.540	2450	1999	El Alaoui et al. 1999
Morocco	High Atlas	Tizrag	31.300	-7.540	2650	2000	Alifriqui 2000
Morocco	High Atlas	Tizrag	31.300	-7.540	-	1993	El Alaoui et al. 1993
Morocco	High Atlas	Toufrine	31.340	-6.920	2150	2008	Personal observation (Romo & Boratyński)
Morocco	High Atlas	Toufrine	31.380	-6.880	2340	2005	Personal observation (Romo, Boratyński & Didukh)
Morocco	High Atlas	Toufrine	31.390	-6.890	2080	2005	Personal observation (Romo, Boratyński & Didukh)
Morocco	High Atlas	Toufrine	31.390	-6.870	2500	2005	Personal observation (Romo, Boratyński & Didukh)
Morocco	High Atlas	Vallée du Zat	31.210	-7.600	2500	1988	Gauquelin & Dagnac 1988
Morocco	High Atlas	Vallée du l'Azzaden	31.100	-7.960	2250	1988	Gauquelin & Dagnac 1988
Morocco	High Atlas	Vallée du Zat	31.320	-7.590	2300	1988	Gauquelin & Dagnac 1988
Morocco	High Atlas	Assif n'Tissouit	32.600	-4.630	2050	1978	Peyre 1978

Morocco	High Atlas	Jbel Aberdouz	32.250	-5.120	2700	2000	Taleb 2000
Morocco	High Atlas	Jbel Afadaï	32.400	-4.770	2400	1978	Peyre 1978
Morocco	High Atlas	Jbel Afadaï	32.410	-4.780	2200	1978	Peyre 1978
Morocco	High Atlas	Jbel Ayachi	32.520	-4.890	2705	1994	Benabid and Fennane 1994
Morocco	High Atlas	Jbel Ayachi	32.530	-4.990	1950	1987	Quézel, Barbero and Benabid 1987
Morocco	High Atlas	Jbel Ayachi	32.530	-4.990	2000	1987	Quézel, Barbero and Benabid 1987
Morocco	High Atlas	Jbel Ayachi	32.620	4.800	1700	1992	Barbero, Benabid, Loisel and Rivas-Mart. 1992
Morocco	High Atlas	Jbel Ayachi	32.620	4.800	1750	1992	Barbero, Benabid, Loisel and Rivas-Mart. 1992
Morocco	High Atlas	Mitkane	32.480	-5.160	2030	1981	Quezel and Barbero 1981
Morocco	High Atlas	Mitkane	32.490	-5.160	1900	1981	Quezel and Barbero 1981
Morocco	High Atlas	Taarirt Adraka	32.400	-4.990	2200	1978	Peyre 1978
Morocco	High Atlas	Taarirt Adraka	32.440	-4.940	2400	1978	Peyre 1978
Morocco	High Atlas	Tizi n'Talghaumt	32.580	-4.570	2180	2011	Personal observation (Romo & Boratyński)
Morocco	High Atlas	Tizi n'Talghaumt	32.580	-4.570	2200	2011	Personal observation (Romo & Boratyński)
Morocco	High Atlas	Tizi n'Talghaumt	32.580	-4.560	2100	2011	Personal observation (Romo & Boratyński)
Morocco	High Atlas	Tizi n'Talghaumt	32.590	-4.570	2100	1992	Barbero, Benabid, Loisel and Rivas-Mart. 1992
Morocco	High Atlas	Tizi n'Talghaumt	32.590	-4.560	2100	2011	Personal observation (Romo & Boratyński)
Morocco	High Atlas	Tizi n'Talghaumt	32.590	-4.550	2000	2011	Personal observation (Romo & Boratyński)
Morocco	High Atlas	Tizi n'Talghaumt	32.600	-4.530	1900	2011	Personal observation (Romo & Boratyński)
Morocco	High Atlas	Tizi n'Talghaumt	32.610	-4.530	1850	1992	Barbero, Benabid, Loisel and Rivas-Mart. 1992
Morocco	High Atlas	Tizi n'Talghaumt	32.610	-4.520	1990	1992	Barbero, Benabid, Loisel and Rivas-Mart. 1992
Morocco	High Atlas	Tounfite	32.410	-5.170	2300	1939	Emberger 1939
Morocco	High Atlas	Tounfite	32.450	-5.270	1800-2150	2002	Gauquelin et al. 2002
Morocco	Middle Atlas	Afnourir (Lac Afenourir)	33.290	-5.260	1830	1988	Benabid 1988
Morocco	Middle Atlas	Afnourir (Lac Afenourir)	33.290	-5.260	1830	1988	Benabid 1988
Morocco	Middle Atlas	Afnourir (Lac Afenourir)	33.290	-5.260	-	1969	Lecompte 1969
Morocco	Middle Atlas	Aguelmane de Sidi Ali	33.080	-5.020	2100	2002	Personal observation (Romo & Boratyński)
Morocco	Middle Atlas	Aguelmane de Sidi Ali	33.076	-5.028	2200	2014	Personal observation (Boratyński, Romo, Dering, Sękiewicz & Sękiewicz)
Morocco	Middle Atlas	Aïn Kahla	33.230	-5.230	1900	1924	Personal observation (Jahandiez)
Morocco	Middle Atlas	Aïn Kahla	33.230	-5.230	1950	1999	El Alauli et al. 1999
Morocco	Middle Atlas	Aïn Kahla	33.230	-5.230		1986	Lecompte 1986

Morocco	Middle Atlas	Aïn Nokra	33.250	-4.930	1800	1999	El Alauli et al. 1999
Morocco	Middle Atlas	Aït Kaïs	33.380	-4.710	1900	1969	Lecompte 1969
Morocco	Middle Atlas	Aquelmane du Sidi Ali	33.050	-5.000	1900	1999	El Alaouli et al..1999
Morocco	Middle Atlas	Aquelmane du Sidi Ali	33.050	-5.000	2100	1924	Personal observation (Jahandiez)
Morocco	Middle Atlas	Aquelmane du Sidi Ali	33.050	-5.000	2260	2005	Personal observation (Romo & Boratyński)
Morocco	Middle Atlas	Aquelmane du Sidi Ali	33.060	-5.030	2200	2005	Personal observation (Romo & Boratyński)
Morocco	Middle Atlas	Aquelmane du Sidi Ali	33.070	-5.030	2220	2005	Personal observation (Romo & Boratyński)
Morocco	Middle Atlas	Aquelmane du Sidi Ali	33.070	-5.000	2120	2005	Personal observation (Romo & Boratyński)
Morocco	Middle Atlas	Aquelmane du Sidi Ali	33.070	-4.990	2110	2005	Personal observation (Romo & Boratyński)
Morocco	Middle Atlas	Aquelmane du Sidi Ali	33.080	-4.980	2280	2005	Personal observation (Romo & Boratyński)
Morocco	Middle Atlas	Aquelmane du Sidi Ali	33.090	-5.030	2100	1988	Gauquelin & Dagnac 1988
Morocco	Middle Atlas	Aquelmane du Sidi Ali	33.090	-5.030	2100	2005	Personal observation (Romo & Boratyński)
Morocco	Middle Atlas	Arbalou Arbi	33.160	-4.870	1850-2200	2002	Gaugueline et al. 2002
Morocco	Middle Atlas	Arbalou Arbi	33.160	-4.870	-	1969	Lecompte 1969
Morocco	Middle Atlas	Azrou	33.080	-5.200	2000	1997	Personal observation (Jury 1997)
Morocco	Middle Atlas	Azrou	33.080	-5.020	2000	2002	Personal observation (Romo & Boratyński)
Morocco	Middle Atlas	Col du Zad	32.960	-5.150	1980	1988	Benabid 1988
Morocco	Middle Atlas	Col du Zad	33.020	-5.080	2200	2004	Personal observation (Romo & Boratyński)
Morocco	Middle Atlas	Feldi	33.230	-4.950	1980	1969	Lecompte 1969
Morocco	Middle Atlas	Izdi Ouareg	33.220	-5.180	2050	1969	Lecompte 1969
Morocco	Middle Atlas	Izdi Ouareg	33.220	-5.180	2050	1999	El Alauli et al.. 1999
Morocco	Middle Atlas	Jbel Ben Ig (Ij)	33.250	-4.970	2400	1969	Lecompte 1969
Morocco	Middle Atlas	Jbel Bou Iblane	33.590	-4.180	1780	2008	Personal observation (Romo & Boratyński)
Morocco	Middle Atlas	Jbel Bou Iblane	33.590	-4.180	1890	2008	Personal observation (Romo & Boratyński)
Morocco	Middle Atlas	Jbel Bou Iblane	33.590	-4.170	1800	2008	Personal observation (Romo & Boratyński)
Morocco	Middle Atlas	Jbel Bou Iblane	33.600	-4.160	1870	2008	Personal observation (Romo & Boratyński)
Morocco	Middle Atlas	Jbel Bou Iblane	33.600	-4.160	1960	2008	Personal observation (Romo & Boratyński)
Morocco	Middle Atlas	Jbel Bou Iblane	33.600	-4.160	2080	2005	Personal observation (Romo, Boratyński & Didukh)
Morocco	Middle Atlas	Jbel Bou Iblane	33.600	-4.150	1920	2008	Personal observation (Romo & Boratyński)
Morocco	Middle Atlas	Jbel Bou Iblane	33.600	-4.150	2000	2008	Personal observation (Romo & Boratyński)
Morocco	Middle Atlas	Jbel Bou Iblane	33.610	-4.160	2200	2005	Personal observation (Romo, Boratyński & Didukh)
Morocco	Middle Atlas	Jbel Bou Iblane	33.610	-4.140	1950	2008	Personal observation (Romo & Boratyński)

Morocco	Middle Atlas	Jbel Bou Iblane	33.610	-4.130	1970	2008	Personal observation (Romo & Boratyński)
Morocco	Middle Atlas	Jbel Bou Iblane	33.620	-4.180	2100	2005	Personal observation (Romo, Boratyński & Didukh)
Morocco	Middle Atlas	Jbel Bou Iblane	33.630	-4.180	2460	2005	Personal observation (Romo, Boratyński & Didukh)
Morocco	Middle Atlas	Jbel Bou Iblane	33.640	-4.170	2300	2005	Personal observation (Romo, Boratyński & Didukh)
Morocco	Middle Atlas	Jbel Bou Iblane	33.640	-4.160	2260	2005	Personal observation (Romo, Boratyński & Didukh)
Morocco	Middle Atlas	Jbel Bou Iblane	33.640	-4.160	2340	2005	Personal observation (Romo, Boratyński & Didukh)
Morocco	Middle Atlas	Jbel Bou Iblane	33.600	-4.160	2000	1981	Personal observation (Ayafi)
Morocco	Middle Atlas	Jbel Bou Iblane	33.710	-4.000	2600	1994	Benabid and Fennane 1994
Morocco	Middle Atlas	Jbel Bou Iblane	33.640	-4.160	2300-2350	2002	Gauquelin et al. 2002
Morocco	Middle Atlas	Jbel Bou Iblane	33.630	-4.180	1920	2013	Romo et al. 2013
Morocco	Middle Atlas	Jbel Bou Mennzel	33.070	-4.960	2360	1969	Lecompte 1969
Morocco	Middle Atlas	Jbel Habbid	33.220	-4.840	2300	1969	Lecompte 1969
Morocco	Middle Atlas	Jbel Hebri	33.270	-5.180	1890	1997	Personal observation (Jury 1997)
Morocco	Middle Atlas	Jbel Hebri	33.320	-5.170	1925	1997	Personal observation (Jury 1997)
Morocco	Middle Atlas	Jbel Irhane	33.200	-4.960	2100	1988	Gauquelin & Dagnac 1988
Morocco	Middle Atlas	Jbel Irhane	33.220	-4.920	2175	1969	Lecompte 1969
Morocco	Middle Atlas	Jbel Irhenba	33.230	-4.900	2200	1969	Lecompte 1969
Morocco	Middle Atlas	Jbel Oukdisou	33.240	-4.810	2250	1969	Lecompte 1969
Morocco	Middle Atlas	Jbel Tajda	33.310	-4.900	2000	1969	Lecompte 1969
Morocco	Middle Atlas	Jbel Tamezziant-Tamokrannt	33.080	-4.790	1900	1969	Lecompte 1969
Morocco	Middle Atlas	Jbel Tichchoukt	33.240	-4.640	2050	1969	Lecompte 1969
Morocco	Middle Atlas	Midelt	33.030	-5.050	2300	1997	Personal observation (Jury 1997)
Morocco	Middle Atlas	Mischliffene	33.410	-5.130	1860	2002	Personal observation (Romo & Boratyński)
Morocco	Middle Atlas	Mischliffene	33.420	-5.110	2000	1999	El Alaoui et al. 1999
Morocco	Middle Atlas	Mischliffene	33.420	-5.110	2000	1988	Gauquelin & Dagnac 1988
Morocco	Middle Atlas	Mischliffene	33.420	-5.110	-	1969	Lecompte 1969
Morocco	Middle Atlas	Sidi Mguild	33.180	-5.180	1960	1988	Benabid 1988
Morocco	Middle Atlas	Sidi Mguild	33.180	-5.180	2000	1999	El Alauoi et al. 1999
Morocco	Middle Atlas	Sidi Mguild	33.180	-5.180	2120	1988	Benabid 1988
Morocco	Middle Atlas	Tamtroucht	33.740	-4.060	2000	1927	Personal observation (Maire 1927)
Morocco	Middle Atlas	Tassamakt	33.210	-5.090	1890	1988	Benabid 1988
Morocco	Middle Atlas	Tassent	32.350	-5.750	1500	1997	Personal observation (Jury 1997)

Morocco	Middle Atlas	Terselt	33.310	4.800	2280	1969	Lecompte 1969
Morocco	Middle Atlas	Tichoukt n'Tifratine	33.420	-4.630	2440	1992	Barbero, Benabid, Loisel and Rivas-Mart. 1992
Morocco	Middle Atlas	Tichoukt n'Tifratine	33.420	-4.630	-	1969	Lecompte 1969
Morocco	Middle Atlas	Tichoukt n'Tifratine	33.430	-4.620	2250	1982	Barbero, Benabid, Loisel & Rivas-Mart. 1982
Morocco	Middle Atlas	Tizi Bou Zabel	33.550	-4.150	2200	2013	Romo et al. 2013
Morocco	Middle Atlas	Tizi n'Taddate	33.090	-4.950	2250	1969	Lecompte 1969
Morocco	Middle Atlas	Tizi n'Tarzeft	33.160	-4.870	2200	1988	Gauquelin & Dagnac 1988
Morocco	Middle Atlas	Tizi n'Tizdi	33.490	-3.920	2200	1978	Peyre 1978
Morocco	Middle Atlas	Tizi n'Tizdi	33.490	-3.910	2000	1978	Peyre 1978
Morocco	Middle Atlas	Tizi n'Tizdi	33.510	-3.940	2600	1978	Peyre 1978
Morocco	Middle Atlas	Tizi n'Tizdi	33.510	-3.890	2250	1978	Peyre 1978
Morocco	Middle Atlas	Tizi n'Tizdi	33.530	-3.930	2500	1978	Peyre 1978
Morocco	Middle Atlas	Tizi n'Tizdi	33.530	-3.930	2400-2500	1978	Peyre 1978
Morocco	Middle Atlas	Tizi n'Tizdi	33.530	-3.910	2370	1978	Peyre 1978
Morocco	Middle Atlas	Tizi n'Tizdi	33.540	-4.140	2400	1978	Peyre 1978
Morocco	Middle Atlas	Tizi n'Tizdi	33.540	-3.950	2300-2400	1978	Peyre 1978
Morocco	Middle Atlas	Tizi n'Tizdi	33.560	-3.950	3600	1978	Peyre 1978
Morocco	Middle Atlas	Tizi n'Tizdi	33.570	-3.940	2400	1992	Barbero, Benabid, Loisel and Rivas-Mart. 1992
Morocco	Middle Atlas	Tizi n'Tizdi	33.570	-3.940	2400	1981	Barbero, Quézel & Rivas-Mart. 1981
Morocco	Middle Atlas	Tizi n'Tizdi	33.570	-3.940	-	2002	Rahou 2002
Spain	Albacete	Nerpio	38.120	-2.400	1310	2016	Mezquida, Rodríguez-García, Olano, 2016
Spain	Albacete	Nerpio	38.150	-2.320	1285	2010	Montesinos et al. 2010
Spain	Albacete	Ossa de Montiel	38.880	-2.770	910	2016	Mezquida, Rodríguez-García, Olano, 2016
Spain	Albacete	Ossa de Montiel	38.900	-2.660	966	2013	Romo et al. 2013
Spain	Albacete	Ossa de Montiel	38.940	-2.270	1000	2010	Montesinos et al. 2010
Spain	Burgos	Alto de Rivarredonda	40.870	-2.290	1030	2012	Gimeno et al. 2012a
Spain	Burgos	Araúzo de Miel	41.860	-3.370	1150	2008	Ponce 2008
Spain	Burgos	Brazacorta	41.700	-3.400	900	2008	Ponce 2008
Spain	Burgos	Carazo	41.988	-3.370	1420	2001	Personal observation (Boratyński & Boratyńska)
Spain	Burgos	Carazo	41.984	-3.350	1325	2001	Personal observation (Boratyński & Boratyńska)
Spain	Burgos	Carazo	41.978	-3.350	1260	2001	Personal observation (Boratyński & Boratyńska)
Spain	Burgos	Carazo	41.976	-3.338	1360	2001	Personal observation (Boratyński & Boratyńska)

Spain	Burgos	Ciruelos de Cervera	41.910	-3.520	1030	2008	Ponce 2008
Spain	Burgos	Contreras	42.010	-3.420	1200	2008	Ponce 2008
Spain	Burgos	Contreras	42.020	-3.430	1100	2008	Ponce 2008
Spain	Burgos	Covarrubias	42.050	-3.500	1020	2013	Romo et al. 2013
Spain	Burgos	Covarrubias	42.070	-3.520	1030	2008	Ponce 2008
Spain	Burgos	Doña Santos	41.880	-3.400	1090	2008	Ponce 2008
Spain	Burgos	Espinosa de Cervera	41.900	-3.460	1085	2008	Ponce 2008
Spain	Burgos	Guma	41.630	-3.520	850	2008	Ponce 2008
Spain	Burgos	Hinojar del Rey	41.760	-3.330	980	2008	Ponce 2008
Spain	Burgos	Hontoria de Rio Franco	42.060	-4.020	850	2008	Ponce 2008
Spain	Burgos	Hontoria del Pinar	41.840	-3.160	1150	2008	Ponce 2008
Spain	Burgos	Hornuez	41.500	-3.630	1130	2010	Montesinos et al. 2010
Spain	Burgos	Hortezuelos	41.930	-3.460	1240	2008	Ponce 2008
Spain	Burgos	Hortigüela	42.053	-3.436	920	2010	Personal observation (Boratyński & Boratyńska)
Spain	Burgos	Hortigüela	42.055	-3.439	990	2010	Personal observation (Boratyński & Boratyńska)
Spain	Burgos	Hortigüela	42.058	-3.443	1060	2010	Personal observation (Boratyński & Boratyńska)
Spain	Burgos	Lerma	41.920	-3.520	1090	2016	Mezquida, Rodríguez-García, Olano, 2016
Spain	Burgos	Navas del Pinar	41.840	-3.200	1210	2008	Ponce 2008
Spain	Burgos	Nebreda	41.950	-3.640	980	2008	Ponce 2008
Spain	Burgos	Retuerta	42.040	-3.510	940	2008	Ponce 2008
Spain	Burgos	San Juan del Monte	41.680	-3.540	860	2008	Ponce 2008
Spain	Burgos	San Pedro de Alanza	42.051	-3.468	975	2010	Personal observation (Boratyński & Boratyńska)
Spain	Burgos	Santa Cruz de Salceda	41.590	-3.570	900	2008	Ponce 2008
Spain	Burgos	Santa Inès	42.050	-3.710	890	2008	Ponce 2008
Spain	Burgos	Santíbañez de Val	41.970	-3.490	990	2008	Ponce 2008
Spain	Burgos	Santo Domingo de Silos	41.960	-3.410	1050	2008	Ponce 2008
Spain	Burgos	Santo Domingo de Silos	41.953	-3.415	1105	2015	Personal observation (Boratyński & Boratyńska)
Spain	Burgos	Santo Domingo de Silos	41.947	-3.400	1130	2015	Personal observation (Boratyński & Boratyńska)
Spain	Burgos	Santo Domingo de Silos	41.943	-3.377	1225	2015	Personal observation (Boratyński & Boratyńska)
Spain	Burgos	Santo Domingo de Silos	41.935	-3.375	1270	2015	Personal observation (Boratyński & Boratyńska)
Spain	Burgos	Santo Domingo de Silos	41.956	-3.390	1100	1999	Personal observation (Boratyński, Didukh, Tomaszewski & Boratyński)

Spain	Burgos	Santo Domingo de Silos	41.967	-3.420	1190	2005	Iszkulo et al. 2011a
Spain	Burgos	Santo Domingo de Silos	41.974	-3.410	1150	2001	Personal observation (Boratyński & Boratyńska)
Spain	Burgos	Santo Domingo de Silos	41.974	-3.390	1280	2001	Personal observation (Boratyński & Boratyńska)
Spain	Burgos	Santo Domingo de Silos	41.972	-3.380	1250	2001	Personal observation (Boratyński & Boratyńska)
Spain	Burgos	Santo Domingo de Silos	41.949	-3.411	1050	2015	Personal observation (Boratyński & Boratyńska)
Spain	Burgos	Santo Domingo de Silos	41.953	-3.446	1020	2015	Personal observation (Boratyński & Boratyńska)
Spain	Burgos	Santo Domingo de Silos	41.949	-3.444	1060	2015	Personal observation (Boratyński & Boratyńska)
Spain	Burgos	Solarana	41.960	-3.660	995	2008	Ponce 2008
Spain	Burgos	Tejada	41.960	-3.530	1130	2008	Ponce 2008
Spain	Castellón	Penyagolosa	40.310	-0.340	1177	2013	Romo et al. 2013
Spain	Castellón	Portell de Morella	40.530	-0.250	1120	2010	Montesinos et al. 2010
Spain	Cuenca	Buenache de la Sierra	40.130	-1.950	1360	2016	Mezquida, Rodríguez-García, Olano, 2016
Spain	Cuenca	Puerto del Rocho	39.890	-1.940	1091	2013	Romo et al. 2013
Spain	Cuenca	Serrania de Cuenca, between La Toba and Buenache de la Sierra	40.160	-1.960	1400	2006	Personal observation (Boratyński & Boratyńska)
Spain	Guadalajara	Ablanque	40.890	-2.220	1050	2012	Gimeno et al. 2012a
Spain	Guadalajara	Buenafuente de Sistal	40.820	-2.210	1220	2012	Gimeno et al. 2012b
Spain	Guadalajara	Cerro de Ablanque	40.890	-2.230	1110	2012	Gimeno et al. 2012a
Spain	Guadalajara	Huertahernando	40.820	-2.280	1140	2012	Gimeno et al. 2012a
Spain	Guadalajara	Mandayona	41.923	-2.740	1070	2007	Personal observation (Boratyński & Boratyńska)
Spain	Guadalajara	Ribarredonda	40.870	-2.300	1020	2012	Gimeno et al. 2012a
Spain	Guadalajara	Río Salado	40.840	-2.300	975	2012	Gimeno et al. 2012a
Spain	Guadalajara	Río Salado	40.850	-2.300	960	2012	Gimeno et al. 2012b
Spain	Huesca	Candasnos	41.360	-0.090	320	2001	Personal observation (Boratyński & Boratyńska)
Spain	Huesca	Candasnos	41.340	-0.060	360	2001	Personal observation (Boratyński & Boratyńska)
Spain	Huesca	Lanaja	41.670	-0.350	1365	2014	Teixeira et al. 2014
Spain	Huesca	Monegros	41.810	-0.550	1000	2014	Teixeira et al. 2014
Spain	León	Barrios de Luna	42.870	-5.850	1170	2016	Mezquida, Rodríguez-García, Olano, 2016
Spain	León	Barrios de Luna	42.860	-5.860	1200	2010	Montesinos et al. 2010
Spain	León	Crémenes	42.920	-5.150	1150	2010	Montesinos et al. 2010
Spain	León	Crémenes	42.920	-5.120	1060	2008	Ponce 2008
Spain	León	Los Barios de Luna	42.853	-5.838	1450	2015	Personal observation (Boratyński & Boratyńska)

Spain	León	Los Barios de Luna	42.878	-5.832	1425	2015	Personal observation (Boratyński & Boratyńska)
Spain	León	Los Barios de Luna	42.890	-5.850	1490	2015	Personal observation (Boratyński & Boratyńska)
Spain	León	Luna	42.880	-5.840	1350	2014	Teixeira et al. 2014
Spain	León	Luna	42.880	-5.860	1239	2014	Teixeira et al. 2014
Spain	León	Minera de Luna	42.885	-5.870	1170	2015	Personal observation (Boratyński & Boratyńska)
Spain	León	Mirantes de Luna	42.860	-5.830	1400	2008	Ponce 2008
Spain	León	Montes Leon	42.833	-5.850	-	2008	Terrab et al. 2008
Spain	Lleida	Alins	42.550	-1.310	1140	2013	Romo et al. 2013
Spain	Lleida	Alins	42.560	1.330	1200	2013	Aymerich and Villar 2013
Spain	Lleida	Alins	42.550	1.330	1200	2013	Aymerich and Villar 2013
Spain	Lleida	Alins	42.560	1.330	1220	2013	Aymerich and Villar 2013
Spain	Lleida	Alins	42.550	1.320	1250	2013	Aymerich and Villar 2013
Spain	Lleida	Alins	42.550	1.320	1280	2013	Aymerich and Villar 2013
Spain	Lleida	Alins	42.550	1.320	1290	2013	Aymerich and Villar 2013
Spain	Lleida	Alins	42.540	1.310	1260	2013	Aymerich and Villar 2013
Spain	Lleida	Alins	42.540	1.290	1250	2013	Aymerich and Villar 2013
Spain	Lleida	Alins	42.540	1.280	1220	2013	Aymerich and Villar 2013
Spain	Lleida	Borente	42.530	1.190	1040	2013	Aymerich and Villar 2013
Spain	Lleida	La Bana	42.530	1.230	1020	2013	Aymerich and Villar 2013
Spain	Lleida	Llavorsi	42.490	1.210	850	2013	Aymerich and Villar 2013
Spain	Lleida	Noris	42.560	1.340	1220	2013	Aymerich and Villar 2013
Spain	Lleida	Noris	42.570	1.340	1350	2013	Aymerich and Villar 2013
Spain	Lleida	Noris	42.570	1.340	1410	2013	Aymerich and Villar 2013
Spain	Lleida	Noris	42.570	1.340	1440	2013	Aymerich and Villar 2013
Spain	Lleida	Rbera de Cardos	42.550	1.240	1350	2013	Aymerich and Villar 2013
Spain	Murcia	Nerpio	38.200	-2.180	1290	2013	Romo et al. 2013
Spain	Palencia	Castrillo de Don Juan	41.780	-4.090	850	2008	Ponce 2008
Spain	Palencia	Cevico Navero	41.870	-4.170	910	2008	Ponce 2008
Spain	Palencia	Cevico Navero	41.860	-4.160	880	1997	Roig et al. 1997
Spain	Palencia	Cobos de Cerrato	42.050	-4.000	820	2016	Mezquida, Rodríguez-García, Olano, 2016
Spain	Palencia	Peña Lampa	42.830	-4.850	1200	2016	Mezquida, Rodríguez-García, Olano, 2016
Spain	Palencia	Velilla de Guardo	42.840	-4.830	1200	2013	Romo et al. 2013

Spain	Segovia	Arevalillo de Cega	41.160	-3.890	1030	2008	Ponce 2008
Spain	Segovia	Cedillo de la Torre	41.430	-3.630	1170	2008	Ponce 2008
Spain	Segovia	Consuegra de Murera	41.260	-3.780	990	2008	Ponce 2008
Spain	Segovia	El Arenal	41.170	-3.780	1150	2008	Ponce 2008
Spain	Segovia	El Guijar	41.550	-3.900	1100	2008	Ponce 2008
Spain	Segovia	Hoces del Duraton	41.250	-3.950	-	2008	Terrab et al. 2008
Spain	Segovia	Maderuelo	41.520	-3.510	990	2008	Ponce 2008
Spain	Segovia	Monte Pradenilla	41.160	-3.680	1120	2014	Vizcaíno-Palomar et al. 2014
Spain	Segovia	Prádena	41.120	-3.710	1160	2013	Romo et al. 2013
Spain	Segovia	Prádena	41.120	-3.690	1200	2008	Ponce 2008
Spain	Segovia	Siguero	41.187	-3.620	1100	2006	Personal observation (Boratyński & Boratyńska)
Spain	Segovia	Siguero	41.170	-3.610	1100	2008	Ponce 2008
Spain	Segovia	Sigueruelo	41.170	-3.650	1140	2016	Mezquida, Rodríguez-García, Olano, 2016
Spain	Segovia	Villovela de Prión	41.110	-4.110	910	2008	Ponce 2008
Spain	Soria	Abejar	41.790	-2.790	1180	2013	Romo et al. 2013
Spain	Soria	Abejar	41.780	-2.790	1300	2008	Ponce 2008
Spain	Soria	Abejar	41.790	-2.770	1100	2014	Teixeira et al. 2014
Spain	Soria	Abioncillo de Catalañazor	41.710	-2.870	1055	2006	Personal observation (Boratyński & Boratyńska)
Spain	Soria	Abioncillo de Catalañazor	41.690	-2.870	1060	2006	Personal observation (Boratyński & Boratyńska)
Spain	Soria	Blacos	41.680	-2.850	1010	2008	Ponce 2008
Spain	Soria	Bocigas de Perales	41.350	-3.350	970	2008	Ponce 2008
Spain	Soria	Cabrejas	41.790	-2.770	1200	2014	Teixeira et al. 2014
Spain	Soria	Cabrejas del Pinar	41.780	-2.830	1250	2016	Mezquida, Rodríguez-García, Olano, 2016
Spain	Soria	Cabrejas del Pinar	41.770	-2.830	1240	2006	Personal observation (Boratyński & Boratyńska)
Spain	Soria	Cabrejas del Pinar	41.780	-2.820	1300	2006	Personal observation (Boratyński & Boratyńska)
Spain	Soria	Cabrejas del Pinar	41.790	-2.830	1230	2006	Personal observation (Boratyński & Boratyńska)
Spain	Soria	Cabrejas del Pinar	41.790	-2.850	1230	2006	Personal observation (Boratyński & Boratyńska)
Spain	Soria	Cabrejas del Pinar	41.780	-2.880	1265	2006	Personal observation (Boratyński & Boratyńska)
Spain	Soria	Cabrejas del Pinar	41.790	-2.870	1340	2006	Personal observation (Boratyński & Boratyńska)
Spain	Soria	Cabrejas del Pinar	41.800	-2.870	1230	2006	Personal observation (Boratyński & Boratyńska)
Spain	Soria	Cabrejas del Pinar	41.770	-2.830	1190	2008	Ponce 2008
Spain	Soria	Cabrejas del Pinar	41.770	-2.850	1130	2006	Personal observation (Boratyński & Boratyńska)

Spain	Soria	Cañon del Rio Lobos	41.717	-3.030	-	2008	Terrab et al. 2008
Spain	Soria	Cañon del Rio Lobos	41.735	-3.040	1100	2006	Personal observation (Boratyński & Boratyńska)
Spain	Soria	Cantalucia	41.760	-2.970	1100	2006	Personal observation (Boratyński & Boratyńska)
Spain	Soria	Castillejo de Robledo	41.550	-3.500	1000	2008	Ponce 2008
Spain	Soria	Castillejo de Robledo	41.560	-3.500	975	2008	Ponce 2008
Spain	Soria	Catalañazor	41.720	-2.830	1100	2010	Montesinos et al. 2010
Spain	Soria	Catalañazor	41.690	-2.830	1030	2008	Ponce 2008
Spain	Soria	Ciria	41.620	-1.930	1118	2016	Mezquida, Rodríguez-García, Olano, 2016
Spain	Soria	Ciria	41.630	-1.960	1080	2008	Ponce 2008
Spain	Soria	Cubilla	41.750	-2.940	1130	2006	Personal observation (Boratyński & Boratyńska)
Spain	Soria	Cubilla	41.730	-2.940	1050	2006	Personal observation (Boratyński & Boratyńska)
Spain	Soria	Fuencaliente de Burgo	41.740	-3.170	1050	2008	Ponce 2008
Spain	Soria	Fuentecantales	41.710	-2.990	1080	2008	Ponce 2008
Spain	Soria	Galapagares	41.440	-2.990	1100	2008	Ponce 2008
Spain	Soria	Herrera de Soria	41.760	-3.030	1110	2008	Ponce 2008
Spain	Soria	Hoz de Arriba	41.380	-3.140	1140	2008	Ponce 2008
Spain	Soria	Iruecha	41.110	-2.110	1260	2008	Ponce 2008
Spain	Soria	Iruecha	41.110	-2.070	1230	2008	Ponce 2008
Spain	Soria	Judes	41.120	-2.180	1250	2016	Mezquida, Rodríguez-García, Olano, 2016
Spain	Soria	Judes	41.120	-2.170	1230	2008	Ponce 2008
Spain	Soria	Judes	41.100	-2.160	1280	2008	Ponce 2008
Spain	Soria	La Cuenca	41.740	-2.730	1120	2008	Ponce 2008
Spain	Soria	La Olmeda	41.550	-3.060	890	2008	Ponce 2008
Spain	Soria	Lodares de Osma	41.570	-3.020	940	2008	Ponce 2008
Spain	Soria	Mosarejos	41.440	-3.030	1070	2008	Ponce 2008
Spain	Soria	Muriel da la Fuente	41.730	-2.860	1070	2008	Ponce 2008
Spain	Soria	Muriel de la Fuente	41.730	-2.850	1060	2006	Personal observation (Boratyński & Boratyńska)
Spain	Soria	Muriel de la Fuente	41.730	-2.860	1020	2006	Personal observation (Boratyński & Boratyńska)
Spain	Soria	Muriel de la Fuente	41.740	-2.860	1085	2006	Personal observation (Boratyński & Boratyńska)
Spain	Soria	Muriel de la Fuente	41.720	-2.890	1040	2006	Personal observation (Boratyński & Boratyńska)
Spain	Soria	Muriel de la Fuente	41.740	-2.840	1120	2006	Personal observation (Boratyński & Boratyńska)
Spain	Soria	Muriel de la Fuente	41.760	-2.840	1125	2006	Personal observation (Boratyński & Boratyńska)

Spain	Soria	Muriel Viejo	41.790	-2.900	1300	2006	Personal observation (Boratyński & Boratyńska)
Spain	Soria	Muriel Viejo	41.780	-2.920	1130	2008	Ponce 2008
Spain	Soria	Nafria de Ucero	41.720	-3.090	1020	2008	Ponce 2008
Spain	Soria	Quintanas Rubias de Abajo	41.450	-3.150	1075	2008	Ponce 2008
Spain	Soria	Rioseco de Soria	41.630	-2.850	1020	2008	Ponce 2008
Spain	Soria	Sabinar de Catalañazor	41.720	-2.840	1010	2006	Personal observation (Boratyński & Boratyńska)
Spain	Soria	Sabinar de Catalañazor	41.720	-2.840	1035	2006	Personal observation (Boratyński & Boratyńska)
Spain	Soria	Sabinar de Catalañazor	41.720	-2.840	1070	2006	Personal observation (Boratyński & Boratyńska)
Spain	Soria	Sabinar de Catalañazor	41.720	-2.840	1110	2006	Personal observation (Boratyński & Boratyńska)
Spain	Soria	Santa María de las Hoyas	41.800	-3.130	1190	2008	Ponce 2008
Spain	Soria	Talveila	41.780	-2.980	1175	2006	Personal observation (Boratyński & Boratyńska)
Spain	Soria	Torremocha Ayllón	41.440	-3.240	1030	2008	Ponce 2008
Spain	Soria	Transecto Alto	40.810	-2.210	1230	2012	Gimeno et al. 2012b
Spain	Soria	Ucero	41.720	-3.060	1030	2006	Personal observation (Boratyński & Boratyńska)
Spain	Soria	Ucero	41.740	-3.030	990	2006	Personal observation (Boratyński & Boratyńska)
Spain	Soria	Ucero	41.740	-3.040	1100	2006	Personal observation (Boratyński & Boratyńska)
Spain	Soria	Ucero	41.760	-3.080	1015	2006	Personal observation (Boratyński & Boratyńska)
Spain	Soria	Ucero	41.780	-3.040	1075	2006	Personal observation (Boratyński & Boratyńska)
Spain	Soria	Ucero	41.750	-3.070	990	2006	Personal observation (Boratyński & Boratyńska)
Spain	Soria	Ucero	41.710	-3.020	1060	2008	Ponce 2008
Spain	Soria	Valdeavillo	41.650	-2.890	1000	2008	Ponce 2008
Spain	Soria	Valdenebro	41.570	-2.960	960	2008	Ponce 2008
Spain	Soria	Valderromán	41.370	-3.130	1240	2008	Ponce 2008
Spain	Soria	Velasco	41.620	-2.980	995	2008	Ponce 2008
Spain	Soria	Villaciervos	41.740	-2.640	1160	2008	Ponce 2008
Spain	Teruel	Barranco de las Columnas	40.320	-1.130	960	2013	Vela, Schäfer 2013
Spain	Teruel	Javalambre, Nava de Torrijos	40.000	-0.980	1177	2013	Romo et al. 2013
Spain	Teruel	Olalla	40.950	-1.170	1120	2016	Mezquida, Rodríguez-García, Olano, 2016
Spain	Teruel	Puerto de Cabigordo near Cedrillas	40.412	-0.940	1500	2001	Personal observation (Boratyński & Boratyńska)
Spain	Teruel	Puerto de Escandon	40.270	-0.980	1240	2016	Mezquida, Rodríguez-García, Olano, 2016
Spain	Teruel	Rubielos de Mora	40.180	-0.630	1060	2006	Personal observation (Boratyński & Boratyńska)
Spain	Teruel	Rubielos de Mora	40.180	-0.620	1200	2006	Personal observation (Boratyński & Boratyńska)

Spain	Teruel	Saldón	40.330	-1.430	1420	2010	Montesinos et al. 2010
Spain	Valencia	El Hontanar	40.030	-0.950	1310	2013	Romo et al. 2013
Spain	Valencia	Puebla de San Miguel	40.050	-1.120	1500	2010	Montesinos et al. 2010
Spain	Zamora	Toro, Duero Basin	41.517	-5.380	740	2008	Terrab et al. 2008
Spain	Zaragoza	Bujaraloz	41.500	-0.050	342	2013	Romo et al. 2013
Spain	Zaragoza	Bujaraloz	41.474	-0.250	400	1996	Personal observation (Boratyński & Boratyńska)
Spain	Zaragoza	Bujaraloz	41.474	-0.250	400	1999	Personal observation (Boratyński, Didukh, Tomaszewski & Boratyński)
Spain	Zaragoza	Calcena	41.650	-1.700	850	2008	Terrab et al. 2008
Spain	Zaragoza	La Riba	40.920	-2.290	1010	2012	Gimeno et al. 2012a
Spain	Zaragoza	Osera del Ebro	41.533	-0.570	300	2008	Terrab et al. 2008
Spain	Zaragoza	Pina de Ebro	41.480	-0.250	360	2016	Mezquida, Rodríguez-García, Olano, 2016
Spain	Zaragoza	Purburrell (Bujaraloz)	41.480	-0.280	350	2010	Montesinos et al. 2010

Supplementary Table S3. Contribution of bioclimatic variables in theoretical current distribution of *J. thurifera* (WorldClim)

**Variable**		**All (%)**	**European-only**	**African-only**
BIO1	Annual Mean Temperature	1.6	1.7	4.6
BIO10	Mean Temperature of Warmest Quarter	2.5	1.0	7.0
**BIO11**	**Mean Temperature of Coldest Quarter**	**28.6**	**17.1**	**31.7**
BIO12	Annual Precipitation	3.2	1.0	4.4
BIO13	Precipitation of Wettest Month	1.4	2.8	0.0
BIO14	Precipitation of Driest Month	2.1	4.2	7.6
BIO15	Precipitation Seasonality	2.7	4.2	0.1
BIO16	Precipitation of Wettest Quarter	0.6	0.5	0.1
BIO17	Precipitation of Driest Quarter	2.1	2.9	0.1
**BIO18**	**Precipitation of Warmest Quarter**	**10.2**	**11.6**	**22.8**
BIO19	Precipitation of Coldest Quarter	0.1	0.1	0.2
BIO2	Mean Diurnal Range	0.2	0.7	0.6
**BIO3**	**Isothermality**	0.2	**11.1**	4.4
BIO4	Temperature Seasonality	0.7	1.8	0.3
BIO5	Max T of Warmest Month	0.3	0.3	3.0
**BIO6**	**Min T of Coldest Month**	**22.8**	**24.0**	5.9
BIO7	Temperature Annual Range	0.3	0.2	0.2
BIO8	Mean Temperature of Wettest Quarter	9.0	7.1	6.0
**BIO9**	**Mean Temperature of Driest Quarter**	**11.4**	**7.7**	1.0

Supplementary Table S4. Contribution of bioclimatic variables in theoretical current distribution of *J. thurifera* (CHELSA)

Variable		All (%)	European-only	African-only
**BIO1**	**Annual Mean Temperature**	8.3	7.6	**13.9**
BIO10	Mean Temperature of Warmest Quarter	3.1	0.4	2.6
**BIO11**	**Mean Temperature of Coldest Quarter**	**25.0**	**17.6**	1.7
BIO12	Annual Precipitation	0.2	0.3	0.1
BIO13	Precipitation of Wettest Month	1.0	1.2	0.1
**BIO14**	**Precipitation of Driest Month**	0.3	3.1	**28.7**
BIO15	Precipitation Seasonality	0.5	1.0	0.3
BIO16	Precipitation of Wettest Quarter	0.3	0.1	0.0
BIO17	Precipitation of Driest Quarter	0.3	13.0	2.2
**BIO18**	**Precipitation of Warmest Quarter**	**16.8**	**16.7**	2.9
BIO19	Precipitation of Coldest Quarter	0.1	0.5	0.1
BIO2	Mean Diurnal Range	2.4	3.7	4.5
BIO3	Isothermality	8.7	3.1	0.4
**BIO4**	**Temperature Seasonality**	1.3	**11.1**	0.7
BIO5	Max T of Warmest Month	1.7	1.0	0.8
**BIO6**	**Min T of Coldest Month**	**21.7**	**10.3**	**38.6**
BIO7	Temperature Annual Range	2.3	5.6	0.6
BIO8	Mean Temperature of Wettest Quarter	4.8	2.8	0.2
BIO9	Mean Temperature of Driest Quarter	1.2	0.9	1.6

Supplementary Table S5. Factor loadings of bioclimatic variables in two first principal component (PC1 – PC1) revealed by the principal component analysis (PCA) (abbreviations of bioclimatic variables as in Supplementary Table S3).

**Variable**	**PC1**	**PC2**
BIO3	**-0.35**	-0.24
BIO6	0.18	**0.39**
BIO8	0.03	**0.56**
BIO9	-0.25	0.02
BIO12	**0.47**	-0.19
BIO13	**0.41**	**-0.32**
BIO15	**-0.31**	**-0.39**
BIO18	**0.42**	0.17
BIO19	**0.35**	**-0.39**

Supplementary Figure S1. Theoretical current range of *J. thurifera*, estimated using MaxEnt (WorldClim); based on European and African records showing current locations (black points). Map prepared with QGIS.

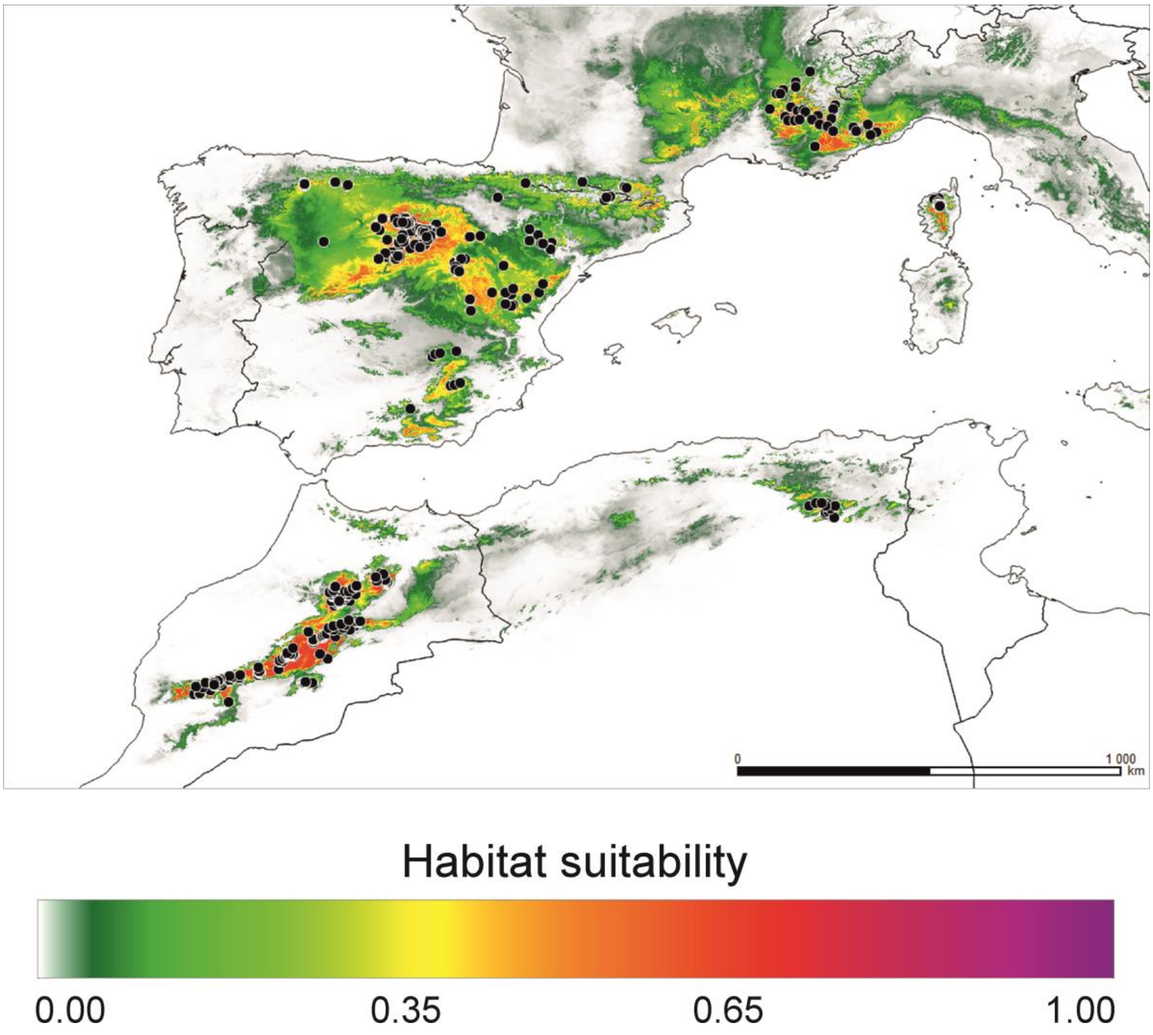

Supplementary Figure S2. Theoretical current range of *J. thurifera*, estimated using MaxEnt based on raster data from WorldClim database: A – European and African records; B – African-only records; C – European-only records. Map prepared with QGIS.

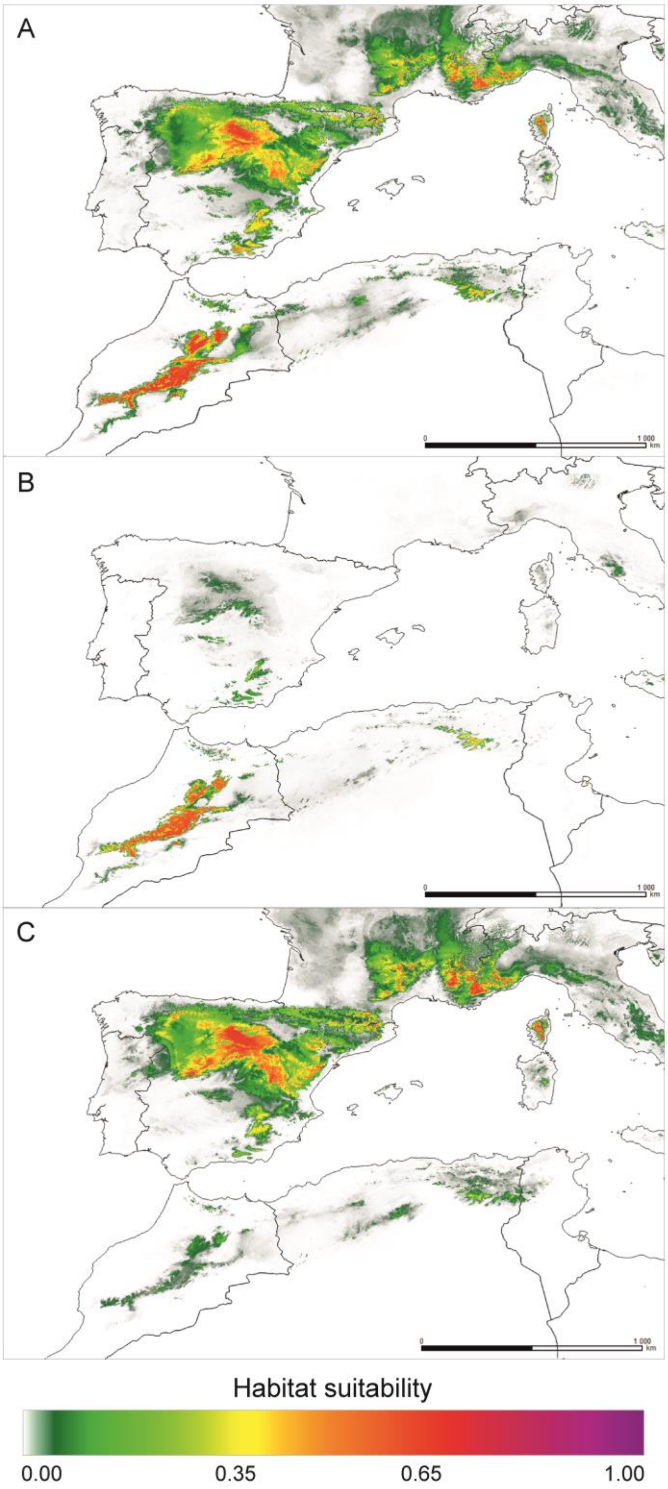

Supplementary Figure S3. Theoretical range of *J. thurifera* in the future estimated using MaxEnt based on raster data from WorldClim database (year 2070). The RCP 2.6 scenario of climate change was used for the CCSM4 model. Map prepared with QGIS (red points habitat suitability ≤ 5%)

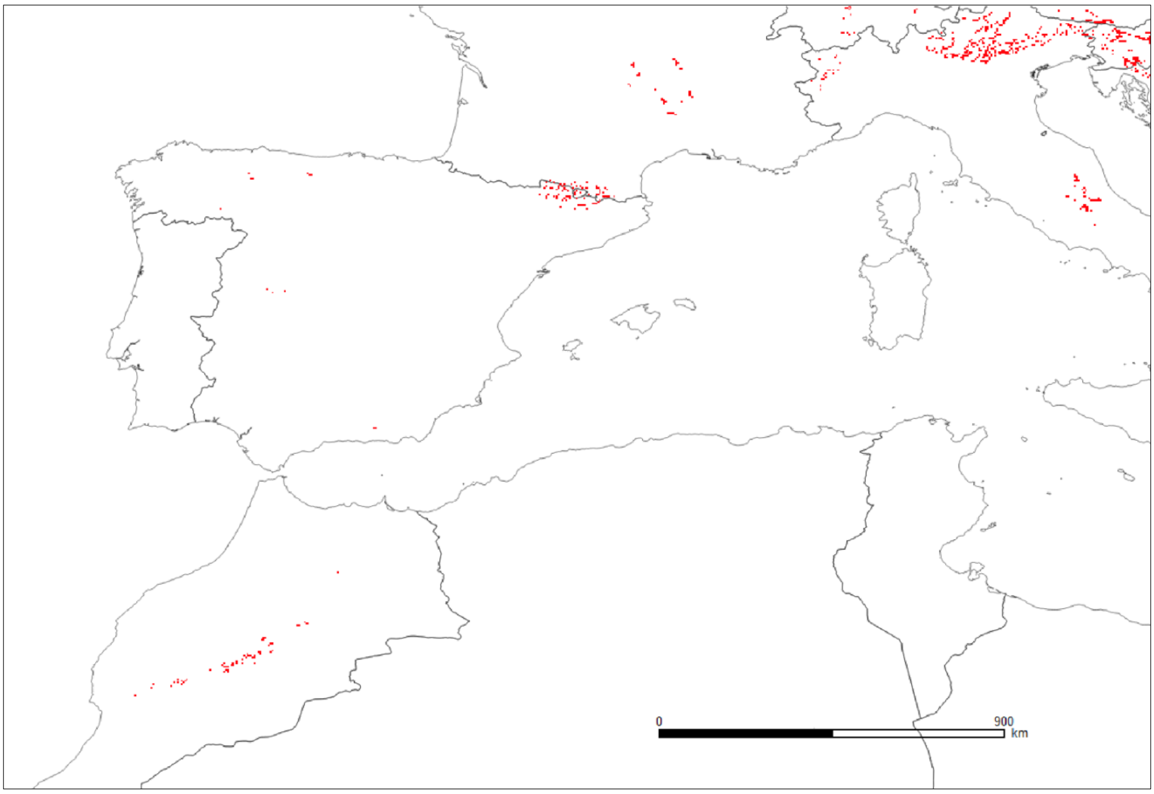

Supplement: Supplementary file 1 — Supplementary information. [file 41598_2020_61525_MOESM1_ESM.pdf]
